# Fibrillarin-mediated 2’-O-methylation serves as a translation brake on uncapped enterovirus RNA

**DOI:** 10.1371/journal.ppat.1014455

**Published:** 2026-07-20

**Authors:** Kui Zhang, Xuliu Zhou, Baocheng Yu, Xiang Guo, Li Zuo, Lishi Liu, Zhen Chen, Xueyan Zhang, Haojie Hao, Huanzhou Xu, Fang Huang, Wuxiang Guan, Haibin Liu

**Affiliations:** 1 Center for Emerging Infectious Diseases, Wuhan Institute of Virology, Center for Biosafety Mega-Science, Chinese Academy of Sciences, Wuhan, Hubei, China; 2 State Key Laboratory of Virology and Biosafety, Wuhan Institute of Virology, Chinese Academy of Sciences, Wuhan, Hubei, China; 3 University of Chinese Academy of Sciences, Beijing, China; 4 School of Pharmacy, Hubei University of Chinese Medicine, Wuhan, Hubei, China; 5 Hubei Jiangxia Laboratory, Wuhan, Hubei, China; Stanford University, UNITED STATES OF AMERICA

## Abstract

2′-O-methylation (Nm) is a key epitranscriptomic mark across diverse RNAs, yet its role in antiviral defense remains largely unexplored. To systematically investigate the landscape and functional significance of internal Nm modifications on enterovirus RNAs, a sensitive sequencing method termed RACE-Nm was established, in combination with the machine learning–based predictor NanoNm, to map Nm sites on low-abundance RNAs at single-nucleotide resolution. A conserved Nm site was identified at nucleotide 307C within the viral internal ribosome entry site (IRES), which was catalyzed by the methyltransferase FBL. The 2′-O-methylation at 307C caused steric hindrance that disrupted PCBP2 recruitment to the IRES and repressed cap-independent translation. Consequently, FBL-mediated suppression of IRES activity limited the production of the viral RNA polymerase 3D, which in turn prevented the upregulation of a set of pro-viral host genes, including FOSL1, ITGB3, and KCNQ4. The physiological importance of this defense mechanism was further demonstrated in vivo, as EV71 mutant lacking the 307C site showed markedly enhanced replication, aggravated tissue damage, and increased lethality in mouse model. Our study establishes FBL-mediated internal Nm modification as a novel host defense pathway that directly targets cap-independent translation to restrict enteroviral infection, highlighting the epitranscriptomic regulation of host–virus interactions.

## Author summary

EV71 causes hand-foot-and-mouth disease and can lead to severe neurological complications in children. It lacks a protective cap on their RNA, making them absent of cap 2′-O-methylation (Nm) modifications. In this study, we found that a human enzyme called Fibrillarin (FBL) places internal Nm on a specific location within the EV71 RNA genome. This tag acts like a brake, physically blocking a crucial host protein (PCBP2) from binding to the translation start sequence. Without this protein, the virus cannot efficiently produce the proteins it needs to replicate. Importantly, when this tag was removed, the virus showed enhanced pathogenesis in mice, causing faster weight loss, higher viral loads, and more severe tissue damage. Our work reveals that Nm is not just a passive modification but an active antiviral mechanism. This suggests new ways to boost this natural brake and fight enterovirus infections.

## Introduction

RNA modifications serve as critical regulators of gene expression. Among them, 2’-O-Methylation (Nm) is the addition of a methyl group to the 2’-hydroxyl group of ribose in all four nucleotide types (A, U, G, C). This modification occurs across diverse RNA species, including mRNA, rRNA, tRNA, snRNA, snoRNA, and miRNA [1,2]. In mRNA, Nm modifications can be classified into two distinct types based on their positional distribution. Cap Nm modifications occur specifically at the first two nucleotides of the 5’ cap structure, and are catalyzed by CMTR1 [3] and CMTR2 [4], respectively. These cap modifications constitute important elements of the 5’ cap structure, prevent mRNA from decapping, degradation, and misrecognition by innate immune systems, and promote translation initiation [1,2]. The second category is internal Nm, which occurs throughout mRNA transcripts but is enriched within CDS regions [5]. This positional preference disrupts key steps of translation elongation to influence protein synthesis [6]. In addition, internal Nm modulate the secondary structure to alter the RNA biological function [7]. Recent studies have identified snoRNAs and the associated methyltransferase fibrillarin (FBL) as the machinery of internal Nm installation [8,9].

The Nm landscape is crucial in the context of viral infection. Many viruses strategically incorporate Nm modifications to evade host immunity and regulate their replication. Nuclear-replicating DNA viruses and retroviruses acquire 5’ cap structures, including cap Nm modifications, by utilizing the host capping machinery [10]. RNA viruses often either employ a cap-snatching mechanism to steal host mRNA caps [11,12], or encode their own capping enzymes. For example, coronaviruses encode both an N7-methyltransferase (nsp14) [13,14] and a 2’-O-methyltransferase (nsp16/nsp10 complex) [15]. The nsp16/nsp10 complex specifically mediates cap Nm formation at the first nucleotide [16], creating a molecular signature that enables evasion of host RNA surveillance systems. Beyond cap Nm modifications, recent study has identified internal Nm sites in HIV RNA mediated by the host 2’-O-methyltransferase FTSJ3 [17]. Notably, FTSJ3 knockdown in HIV-infected cells leads to both decreased viral RNA Nm levels and subsequent activation of type I interferon responses [17]. These findings collectively demonstrate that Nm modifications, either at cap structures or internal positions, are strategically incorporated into viral RNAs to precisely regulate replication efficiency.

The enterovirus genus within the Picornaviridae family contains numerous clinically significant pathogens that cause a spectrum of human diseases [18]. Their genome consists of a ~ 7400 nt positive-sense, single-stranded RNA with a polyadenylated 3’ end but lacks the canonical 5’ cap [19]. Instead, it contains a highly structured 5’ untranslated region (5’-UTR) of approximately 750 nt [20,21]. The 5’-UTR comprises two functional domains: First, a cloverleaf-like structure (5’CL; approximately 1–90 nt) at the 5’ end that serves as a replication platform and assembles viral and host proteins required for genome replication [19]. Second, an internal ribosome entry site (IRES; approximately 90–750 nt) that facilitates cap-independent translation initiation [22,23]. This unique RNA architecture enables the coordinated regulation of viral translation and RNA synthesis. Enterovirus 71 (EV71), the primary causative agent of hand-foot-and-mouth disease, poses a critical public health threat due to its significant neurovirulence and recurrent outbreaks [24]. The life cycle of EV71 is governed by key viral proteins, among which the structural protein VP1 forms the virion’s outer shell to determine serotype and facilitate receptor binding [25], while the non-structural protein 3D functions as the RNA-dependent RNA polymerase, responsible for replicating the viral genome [26]. While previous studies, including our own, have established roles for other RNA modifications (e.g., m6A, m5C, ac4C) in EV71 replication [27–29], the existence and function of internal Nm modifications remain completely unknown.

In this study, we investigated the role of host-derived Nm modifications in regulating EV71 replication and pathogenesis. To this end, the newly developed RACE-Nm, a low-dNTP–based sequencing method, was systematically employed, in combination with complementary approaches including UHPLC-MS/MS, nanopore sequencing-based prediction, and RNA immunoprecipitation. This integrated strategy enabled the systematic identification of internal Nm modifications on the EV71 RNA genome and determined FBL as the responsible methyltransferase. Furthermore, we elucidated the functional significance of a specific Nm site in modulating IRES-dependent translation and established the mechanistic basis for this regulation. Finally, the critical role of this Nm-mediated restriction was confirmed by enhanced viral pathogenesis in a mouse model. Our findings not only reveal a novel antiviral role of FBL-mediated 2’-O-methylation but also highlight the potential of targeting this pathway for therapeutic intervention against EV71 infection.

## Methods

### Virus and cells

The enterovirus 71 (EV71) strain BrCr-TR (CSTR:16533.06.IVCAS06.6305) and strain XF (CSTR:16533.06.IVCAS06.6093) were sourced from National Virus Resource Center in Wuhan. The Vero (ATCC, Manassas, VA, USA; # CCL-81), RD (ATCC; # CCL136), and HEK293T (ATCC; # CRL-11268) cell lines were cultured in Dulbecco’s modified Eagle medium (DMEM; Gibco) containing 10% fetal bovine serum (FBS) at 37°C with 5% CO_2_.

EV71 was propagated in Vero cells using DMEM supplemented with 2% fetal bovine serum (FBS) for one to two days in a Class II biosafety laboratory. The viral titer was determined using the 50% tissue culture infectious dose (TCID₅₀) assay. Briefly, Vero cells were seeded in 96-well plates and cultured overnight until reaching approximately 70% confluence. The following day, the virus culture was serially diluted (10^−1^ to 10^−9^) in DMEM, and each dilution was added to 10 replicate wells (100 µL per well). The plates were then incubated at 37°C with 5% CO_2_. The TCID₅₀ was calculated based on the infection rate at each dilution using the Reed-Muench method [30]: Briefly, logID₅₀ = log(dilution with >50% positive) + PD × (−log dilution factor), where PD = (% positive above 50% − 50%)/ (% positive above 50% − % positive below 50%). The virus titer (TCID₅₀/mL) was then calculated as 1/ (logID₅₀/ viral inoculum volume in mL).

### Plasmid construction and mutagenesis

The FLAG-FBL, FLAG-VP1, and FLAG-3D plasmids were generated by cloning the respective coding sequences (CDSs) into the PXJ40-FLAG vector. For the EV71 5’UTR-eGFP reporter plasmids, the 5′ untranslated region (5’UTR, 1–743) of EV71 was inserted between the cytomegalovirus (CMV) promoter and the enhanced green fluorescent protein (eGFP) gene in the pEGFP-N1 backbone (Clontech). Site-directed mutagenesis was performed to introduce Nm mutations into both the 5’UTR-eGFP reporter plasmids and the infectious EV71 clone (provided by Prof. Bo Zhang, Wuhan Institute of Virology). All constructs were verified by sequencing, and the primers used for plasmid construction are listed in S5 Table.

### In vitro transcription and transfection of EV71 genomic RNA

Infectious EV71 genomic RNA was synthesized by in vitro transcription from a HindⅢ-linearized full-length cDNA clone, using the MEGAscript T7 Kit (#AM1334, Ambion, Austin, TX, USA). Electroporation of the RNA transcript was then performed using a Bio-Rad GenePulser Xcell system in Ingenio Electroporation Solution. Viruses were harvested upon the appearance of cytopathic effects.

### siRNA and plasmid transfection

All siRNAs were designed and synthesized by Tsingke Biotechnology Co., Ltd. (Beijing, China), with their sequences listed in S5 Table. For siRNA transfection, cells were transfected using TransIT-X2 Dynamic Delivery System (#MIR 6005, Mirus Bio) following the manufacturer’s protocol. For plasmid transfection, Lipofectamine 2000 (Invitrogen, Cat# 11668–019) was used according to the manufacturer’s instructions**.**

### qPCR analysis

Gene expression was quantified by SYBR Green-based quantitative real-time PCR (qPCR). Total RNA (800 ng) was reverse transcribed into cDNA using HiScript II 1st Strand cDNA Synthesis Kit (Vazyme Biotech, China; # R211-01). qPCR reactions were performed in triplicate using Hieff qPCR SYBR Green Master Mix (# 11201ES03, Yeasen Biotechnology, China) with gene-specific primers list in S5 Table. Primer specificity through melt curve analysis and the amplification efficiency was between 90% and 110%. Relative gene expression levels were calculated using the comparative threshold cycle (ΔΔCT) method, with normalization to GAPDH. All reactions were performed on a CFX Connect real-time PCR system (BioRad Laboratories).

### RNA-seq and bioinformatics analysis

Total RNA was isolated from EV71 infected cells or siRNA-transfected cells using TRIzol reagent (Thermo Fisher Scientific, Cat# 15596018). RNA-seq was then performed on the DNBSEQ platform (BGI-NGS-JK-RNA-001) as described previously [31]. For library preparation, mRNA was enriched via oligo(dT) magnetic bead-based selection, fragmented, and subjected to quality control. First- and second-strand cDNA synthesis was performed, followed by 3’ adenylation and adapter ligation. For sequencing, circularized DNA was amplified via rolling circle amplification to produce DNA nanoballs (DNBs), which were loaded onto patterned nanoarrays. Combinatorial Probe-Anchor Synthesis (cPAS) technology (DNBSEQ platform, BGI) was employed for 150 bp paired-end sequencing. Raw reads were quality-filtered using SOAPnuke to remove low-quality bases and adapters. Clean reads were mapped to the human reference genome (GRCh38/hg38) using HISAT2. Gene expression levels were estimated with RSEM (v1.3.1) and normalized to FPKM values. Differential Expression analysis was performed using DESeq2 (v1.4.5) with thresholds of |log2FC| ≥ 1 and adjusted p-value < 0.05. Enriched KEGG pathways were identified via Hypergeometric testing (Phyper), with significance thresholds set at Q-value ≤ 0.05 after false discovery rate (FDR) correction. All analyses were conducted using the BGI Tom Multi-omics Platform (https://biosys.bgi.com).

### Western blot

Cells were harvested and lysed in ice-cold lysis buffer (Beyotime, #P0013) for 30 min. The lysates were then centrifuged at 14,000 × g for 10 min at 4°C to remove debris. The cleared supernatants were collected, mixed with loading buffer, and denatured by boiling at 100°C for 10 min. Protein samples were resolved by SDS-PAGE and transferred to nitrocellulose membranes. The membranes were probed with the following primary antibodies: anti-FBL (ABclonal, #A0850), anti-GFP (Proteintech, #66002–1-Ig), anti-β-actin (Santa Cruz, #sc-47778), anti-VP1 (GeneTex, #GTX132338) and anti-3D antibodies (ABclonal, #A8608). After incubation with HRP-conjugated secondary antibodies, protein bands were visualized using a ChemiDoc MP imaging system (Bio-Rad) and quantified with ImageJ software.

### Ultra-high performance liquid chromatography-tandem mass spectrometry (UHPLC-MS/MS)

The purified EV71 virion RNA was prepared as described previously [27,28]. Additionally, EV71 virion RNA was isolated and purified from RD cells transfected with siFBL #1. RNA concentrations were determined using Qubit fluorometric quantification. For enzymatic digestion, 1 μg RNA was mixed with 3 μL buffer, 2 μL S1 nuclease, 1 μL alkaline phosphatase, and 2 μL phosphodiesterase, then brought to 30 μL total volume with nuclease-free water. After brief vortex mixing and centrifugation (10 sec, RT), samples were digested at 37°C for 3 h. The reaction was then diluted with 170 μL nuclease-free water and extracted with 200 μL chloroform (3 min vortex, 5 min centrifugation at 12,000 rpm, RT). The aqueous phase was collected and analyzed using an UPLC-ESI-MS/MS system (UPLC, ExionLC AD, https://sciex.com.cn/; MS, Applied Biosystems 6500 Triple Quadrupole, https://sciex.com.cn/). RNA modifications contents were detected by MetWare (http://www.metware.cn/) based on the AB Sciex QTRAP 6500 LC-MS/MS platform. Significantly regulated metabolites between groups were determined by absolute Log_2_FC (fold change).

### Formaldehyde-crosslinked RNA-immunoprecipitation (RIP)

For each sample, RD cells from two 10-cm plates at 95% confluency (approximately 10⁷ cells per plate) were crosslinked in PBS containing 1% methanol-free formaldehyde for 10 min at 37°C, followed by quenching with 0.125 M glycine. After three ice-cold PBS washes, cells were scraped, pelleted (800 × g, 3 min, 4°C), and lysed in 400 µL RIP buffer (150 mM KCl, 25 mM Tris-HCl pH 7.4, 5 mM EDTA, 0.5 mM DTT, 0.5% NP-40, 100 U/mL RNase inhibitor, 100 µM PMSF, 1 µg/mL protease inhibitors). Cleared lysates (14,000 × g, 10 min) were subjected to overnight IP at 4°C with anti-FBL (Abclonal, #A0850) or rabbit IgG control, followed by 1 h incubation with pre-blocked Protein A beads. Beads were washed three times with high-salt wash buffer (300 mM KCl, 25 mM Tris–HCl pH 7.4, 5 mM EDTA, 0.5 mM DTT, 0.5% NP40, 100 U/ml RNase inhibitor, 100 µmol/L PMSF, 1 μg/mL protease inhibitors) and three times with RIP buffer, after which RNA was extracted using TRIzol post-proteinase K digestion for qPCR analysis of EV71 RNA.

### Nanopore direct RNA sequencing (DRS-seq)

Total RNA was extracted from EV71 (strain XF) infected cells using TRIzol, followed by poly(A) mRNA isolation using the GenElute mRNA Miniprep Kit (Sigma-Aldrich). Direct RNA sequencing was performed according to the Oxford Nanopore DRS protocol (SQK-RNA002) as described previously [28]. Briefly, Poly(A)-selected RNA libraries were prepared and sequenced on a MinION device (Oxford Nanopore Technologies) using a FLO-MIN106D flow cell for 72 hours. Raw multi-fast5 reads were base-called using Guppy (v3.1.5) and converted to single-read fast5 files using the ont_fast5_api (v3.0.2) multi_to_single_fast5 command. Reads were then mapped to the EV71 reference genome (GenBank # JQ804832.1) using Tombo (v1.5.1).

### NanoNm analysis

NanoNm is a machine learning-based tool developed for detecting Nm modifications [9]. This method leverages the distinct electrical signal features of Nm modifications in Nanopore direct RNA sequencing data. The analytical pipeline was established using rRNA datasets with known Nm modifications and in vitro transcripts lacking modifications as background controls. Due to incompatibility with the latest POD5-format nanopore data, we reanalyzed our three previous FAST5-format datasets from an EV71-XF strain [28]. Initial data processing began with converting multi-read fast5 files into single-read format using multi_to_single_fast5, followed by basecalling with Guppy (v6.0.1) using the RNA high-accuracy configuration (rna_r9.4.1_70bps_hac.cfg). The raw current signals were then aligned to reference sequences using Tombo’s resquiggle algorithm with RNA-specific parameters, including global scale fitting and event standard deviation calculation. Feature extraction was performed on individual transcripts using custom Python scripts from the NanoNm package (v1.0.0), processing clipped signal data (5’ end trimming) to capture modification-related signal characteristics. The machine learning prediction phase combined all extracted features into composite files (FASTA and TSV formats) for model application. The trained XGBoost classifier was then applied to predict Nm sites across the EV71 transcriptome using the reference genome (EV71-XF, GenBank # JQ804832.1) and gene-transcript mapping file as annotation inputs. We extracted 5-nucleotide genomic segments (2 bp upstream and downstream of each Nm-modified base) from the reference genome. For each flanking position (-2, -1, + 1, + 2 relative to the central modified nucleotide), we calculated the observed frequencies of all four ribonucleotides (A, U, C, G) across all modification sites. Sequence patterns were visualized using position-weight matrices and sequence logos (ggseqlogo R package), with the central modified position held invariant in all alignments.

### Low-dNTP primer extension

RNA probe (P-2’O, HIV RNA 733–832 nt) with or without Am783 (position 51 nt) and an artificial Cm (position 21 nt) sites, and FAM-labeled RT primer (sequence is listed in S5 Table) were synthesized from Tsingke Biotechnology Co., Ltd.

For low-dNTP primer extension assays [17], 30 ng of RNA oligonucleotide (P-2′O) was annealed with 10 µM FAM-labeled reverse transcription primer in AMV Reverse Transcriptase Reaction Buffer (NEB, M0277S) in a total volume of 5 µL. The mixture was denatured at 96°C for 40 s, chilled on ice for 1 min, incubated at 65°C for 10 min, and again placed on ice for 1 min. Reverse transcription was carried out in a final volume of 5 µL containing 1 mM (high dNTP) or 0.004 mM (low dNTP) dNTPs and 0.5 U of AMV Reverse Transcriptase (NEB, M0277S). Reactions were incubated at 42°C for 1 h and terminated by adding 4 µL of stop solution (95% formamide, 20 mM EDTA, 0.05% bromophenol blue, 0.05% xylene cyanol). Products were denatured at 96°C for 2 min, resolved on 7% TBE-urea polyacrylamide gels, and visualized using an Amersham Typhoon imager (GE Healthcare).

Applying this method to EV71 RNA (BrCr-TR), we replaced FAM-labeled primers with [γ-32P]-labeled primers to enhance method sensitivity. RT primers were radiolabelled with γ32P-ATP using T4 polynucleotide kinase. Subsequently, 300 ng of RNA polyA purified from total RNA of RD cells infected with EV71 was hybridized with 32P-radiolabelled RT primer (10μM) in AMV Reverse Transcriptase Reaction Buffer (NEB, M0277S) in a total volume of 5 µl. Low-dNTP Primer extension was performed as described above and the products were denatured at 96 °C for 2 min and loaded onto a 7% TBE-acrylamide-urea sequencing gel. exposed overnight on a PhosphorImager plate and scanned using an Amersham Typhoon imager (GE Healthcare).

### TA cloning and RACE-Nm

To determine cDNA ends from the low-dNTP primer extension, PCR products were cloned into TA vectors and sequenced as follows. Following primer extension (85°C for 5 min), cDNA was purified using a gel extraction kit (Omega) and poly(C)-tailed using terminal transferase (TdT, M0315S, NEB). Primary PCR amplification was performed using an adaptor-oligo(dG) primer (5’-agtctcgatttcggaGGGGGGGGGGGGGGG-3’) with gene-specific reverse primers. Secondary nested PCR employed the adaptor primer (5’-agtctcgatttcgga-3’) with nested gene-specific primers. PCR products were gel-purified (Omega gel extraction kit) and cloned into the pGEM-T Easy Vector System (Promega) for Sanger sequencing.

Alternatively, specific PCR products were sent to Sangon Biotech (Shanghai, China) Co., Ltd. for sequencing library preparation and sequencing. Briefly, Library preparation was performed by two step PCR. First-round PCR was performed in a 25 μL reaction volume containing 2 μL of DNA template (10 ng/μL), 1 μL each of forward and reverse primer mixes (10 μM), and 15 μL of 2 × Kapa HiFi Ready Mix. Thermal cycling was carried out on a Bio-Rad T100 instrument as follows: initial denaturation at 98°C for 5 min; 8 cycles of 98°C for 30 s, 60°C for 30 s, and 72°C for 30 s; and a final extension at 72°C for 5 min, followed by a 4°C hold. PCR products were analyzed by electrophoresis on 1% (w/v) agarose gels in TBE buffer stained with SYBR Green I and visualized under UV light. Amplicons were then purified using AMPure XP beads. Second-round PCR was conducted in a 30 μL reaction volume containing 2 μL of purified DNA, 1 μL each of index-tagged universal P7 and P5 primers (10 μM), and 15 μL of 2 × Kapa HiFi Ready Mix. The cycling conditions were: 98°C for 3 min; 5 cycles of 94°C for 30 s, 55°C for 20 s, and 72°C for 30 s; and a final extension at 72°C for 5 min. The resulting products were again purified with AMPure XP beads, quantified, and pooled. Paired-end sequencing was performed on an Illumina NovaSeq 6000 or MiSeq platform (PE300 mode).

To identify termination sites characteristic of 2’-O-methylation, raw paired-end reads were first filtered by fastp (v0.23.2) and merged using FLASH (v1.2.11, -p 33 -m 12 -M 300 -x 0.25 -z) to create longer contiguous sequences. The merged reads were then filtered to select those containing the target adapter sequence “agtctcgatttcggaGGGGGGGG”, allowing for one nucleotide mismatch to account for potential sequencing errors while maintaining specificity for Nm-detection products. Adapter sequences and any downstream polyG tracts were subsequently trimmed using Cutadapt to isolate the authentic viral sequences. The processed reads were then aligned to the EV71 reference genome (BrCr-TR, GenBank # U22521.1) using Bowtie2 (v2.2.6). The alignment reads were visualized in IGV. Reverse transcription termination sites were specifically identified as Nm positions. The Nm modification rate was calculated as the ratio of reads containing specific termination sites to the total mapped reads. Sites with a rate greater than 1% were thereby defined as modified.

### RNA oligo pulldown

For RNA pulldown assays, 293T cells transfected with plasmids encoding Flag-PCBP2, Flag-PCBP2-KH1/2 or Flag-PCBP2-KH3. At 48 hours post-transfection were lysed in RIPA buffer (50 mM Tris-HCl, pH 8.0, 150 mM NaCl, 5 mM EDTA, 0.1% SDS, 1.0% Nonidet P-40, 0.5% sodium deoxycholate) supplemented with protease inhibitors. Clarified lysates were incubated with 5’-biotin-conjugated RNA oligonucleotides (307C: 5’-biotin-GCUCAGCACUCCCCCCGUGUAGAUC-3’; 307 Cm: 5’-biotin-GCUCAGCACUCCCCCmCGUGUAGAUC-3’, synthesized by Tsingke Biotechnology) for 2 hours at 4°C. The RNA-protein complexes were then captured by incubation with NeutrAvidin Agarose Beads (Thermo Fisher Scientific, 29200) overnight at 4°C with rotation. After extensive washing with 1 × TBS (Beyotime, ST661) for three times, bound proteins were eluted in 2 × Laemmli Sample Buffer (Bio-Rad, 160737) at 95°C for 10 minutes and analyzed by western blotting.

### EMSA (electrophoretic mobility shift assay)

PCBP2-FLAG was overexpressed in 293T cells. Cells were washed twice with PBS and lysed in lysis buffer containing protease inhibitors on ice for 30 min. The lysate was centrifuged at 12,000 × g for 10 min at 4°C, and the supernatant was incubated with anti-Flag affinity gel overnight at 4°C. The gel was washed three times with TBS (5 min each wash on a shaker at 4°C), and bound proteins were eluted with 3 × Flag peptide (150 μg/ml) for 4 h at 4°C with gentle shaking. The eluted Flag-tagged PCBP2 protein was collected by centrifugation at 6,000 × g for 30 s.

The 5′-FAM-labeled RNA probes (listed in S5 Table, Tsingke Biotechnology) containing either unmodified 307C or 2′-O-methylated 307 Cm were denatured at 65°C for 5 min and slowly cooled to room temperature. Binding reactions (30 μl) were performed in binding buffer containing 10 mM Tris-HCl (pH 7.5), 50 mM KCl, 1 mM EDTA, 0.05% Triton X-100, 5% glycerol, 1 mM DTT, and 40 U/ml RNasin. Each reaction contained 2 μl of FAM-labeled RNA probe (100 μM stock diluted 100-fold to 1 μM) and 8 μl of purified PCBP2-FLAG protein at concentrations of 0.20, 0.15, and 0.10 mg/ml, with buffer replacing protein as a negative control. Binding was carried out at 4°C for 30 min. The RNA-protein complexes were resolved on a 4% native polyacrylamide gel in 0.5 × TBE (inner chamber) and 1 × TBE (outer chamber) at 90 V for 45 min on ice. Fluorescence signals were visualized using a ChemiDoc MP imaging system (Bio-Rad).

### Mouse infection and pathogenesis

Animal experiments were performed following established protocols in our previous publication [28,29]. Briefly, AG6 mice received intraperitoneal injection of 10⁴ PFU of wild-type or Nm mutant EV71 in 50 µL DMEM. Body weights were recorded daily to monitor morbidity. At three days post-infection, mice were euthanized, and tissue samples including brain, intestines, and thigh muscles were collected for subsequent analysis. Viral RNA loads in harvested tissues were quantified by qRT-PCR. For histopathological assessment, tissue specimens were fixed, paraffin-embedded, sectioned, and stained with hematoxylin and eosin. Viral antigen distribution was evaluated by immunohistochemistry using an anti-VP1 antibody, followed by HRP-conjugated secondary antibody and DAB chromogenic detection. All histology and IHC services were provided by Wuhan BioqianDu Biotechnology Co., Ltd.

## Results

### Single-nucleotide resolution identification of EV71 internal Nm sites

**Fig 1:**
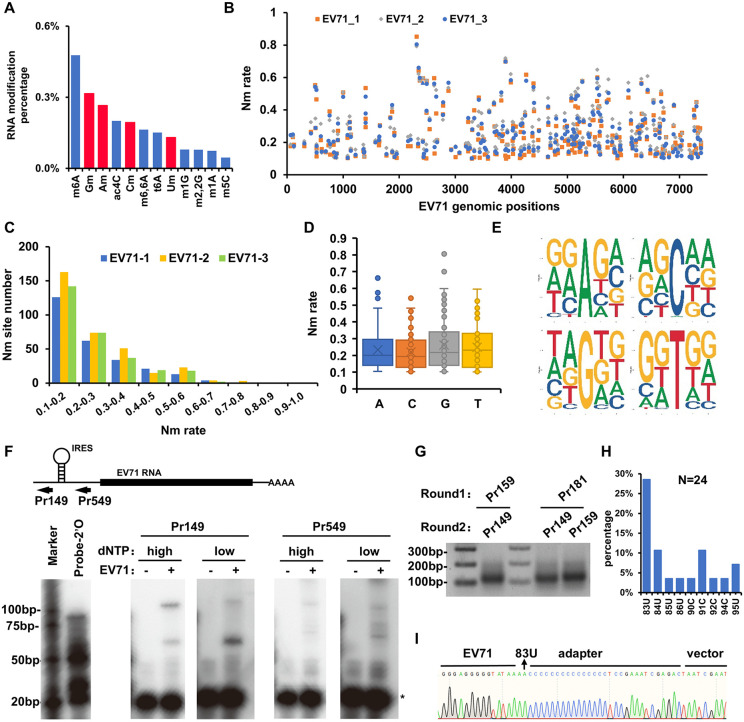
Widespread internal Nm sites in the EV71 genome. **(A)** UHPLC-MS/MS quantification of RNA modifications in purified EV71 virions, showing presence of Gm, Am, Cm, and Um modifications. **(B-E)** Detection of the internal Nm modifications in EV71 RNA by NanoNm. **(B)** Genome-wide distribution of the consistent Nm sites (n = 251) mapped to EV71 RNA. **(C)** Histogram of Nm modification rates (binned 0.1-1.0) in triplicate virus infected samples. **(D)** Box plot comparison of Nm modification frequencies by base type (G/A/C/U). **(E)** Position-weight matrix of nucleotide frequencies flanking Nm sites (-2 to +2 positions). **(F)** Detection of the internal Nm modifications in EV71 RNA by low-dNTP primer extension analysis. EV71-specific detection strategy using [γ-32P]-labeled primers (Pr149 and Pr549). Autoradiograph shows discrete termination band with Pr149; smear pattern with Pr549, indicative of multiple Nm sites. **(G-I)** Validation of the termination products mapped to position 83U. Two-round (nested) PCR strategy was used to amplify 5’ RACE products. **(G)** The first round of primer extension and PCR used primers Pr159 or Pr181. The resulting products were then used as templates for a second round of nested PCR with primers Pr149, or Pr149 and Pr159, respectively. **(H)** Agarose gel electrophoresis of the final PCR product. Bar graph showing the percentage of identified termination sites from sequencing 24 TA clones generated from the nested PCR products. **(I)** A representative Sanger sequencing chromatogram confirming the RT termination site at 83U.

To determine the epitranscriptomic landscape of the EV71 genome, the presence of Nm modifications on the EV71 RNA genome was assessed by using UHPLC-MS/MS, which identified Nm as the most abundant modification (Fig 1A). As the virus lacks a cap structure, these Nm sites were considered internal. To map Nm sites, we employed NanoNm, a machine learning-based tool for Nm site identification and quantification using nanopore direct RNA sequencing data [9]. Applying the same methylation rate cutoff [9], 251 high-confidence Nm sites were identified across replicates (S1A Fig and Fig 1B, S1 Table). Nm rates ranged from 0.1 to 1, with most sites falling between 0.1–0.4 and only a few exceeding 0.6 (Fig 1C). However, no strong base preference was observed among the Nm modifications (Fig 1D). Flanking sequence frequency analysis revealed potential methylation-associated sequence patterns (Fig 1E). Together, these results demonstrate that internal Nm modifications are widespread across the EV71 RNAs and may play a role in regulating viral RNA function.

NanoNm is susceptible to false positives due to inherent nanopore signal noise, raising concerns about its specificity. To precisely map the Nm sites, a method based on reverse transcription (RT) stalling at low dNTP concentrations was developed based on the principle previously used to map Nm in rRNAs and piRNAs [32,33]. A synthetic HIV RNA probe with known Nm sites was used to validate the assay [17] (S1B Fig). Low-dNTP primer extension produced specific termination bands at the expected positions, which were absent in unmodified controls (S1C and S1D Fig). Subsequent 5’ RACE and TA cloning confirmed RT stalling at the modified sites (S1E Fig). This method was then adapted for EV71 RNAs. To enhance sensitivity, virus-specific primers were radiolabeled with [γ-32P]. Primer extension revealed distinct stalling patterns, and nested RACE-PCR identified a predominant Nm site at 83U (Fig 1F–1I).

**Fig 2:**
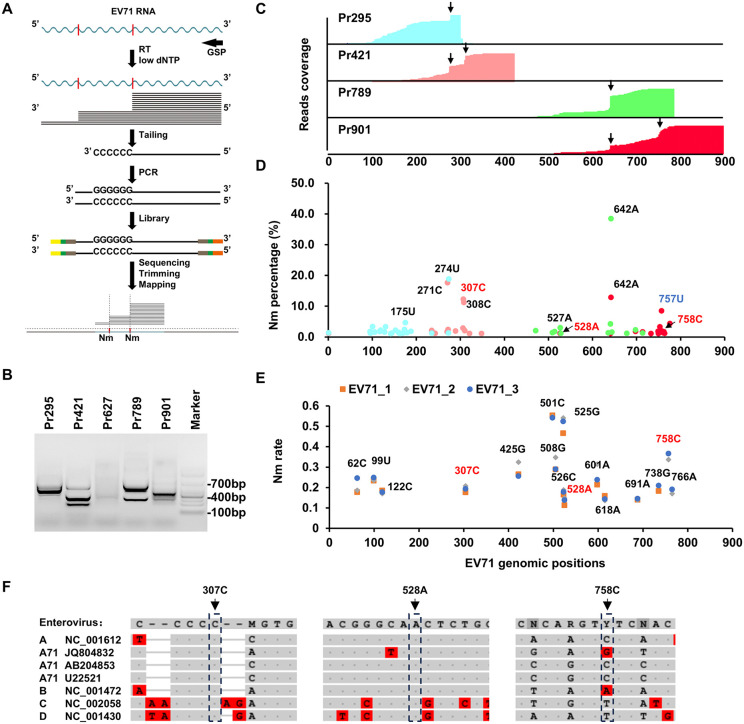
Systematic mapping of EV71 Nm sites by RACE-Nm. **(A)** Experimental workflow includes Low-dNTP reverse transcription of EV71 RNA, Poly(C) tailing of cDNA products, adapter-based PCR amplification, and Target-enriched library preparation for high-throughput sequencing. **(B)** Five primers were designed spanning the EV71 5’UTR. Agarose gel analysis of PCR-amplified termination products. **(C)** IGV visualization of aligned reads showing characteristic 5’ truncation patterns (arrows) at Nm-modified positions. (D) 5’UTR distribution of the Nm sites with a threshold of Nm rate more than 1%. High score or consensus sites are highlighted. **(E)** Genome coordinate conversion of NanoNm-predicted sites (EV71-XF strain, GenBank # JQ804832.1) to BrCr-TR reference (GenBank # U22521.1). **(F)** Evolutionary conservation analysis of identified Nm sites across multiple enterovirus species.

To profile Nm modifications within the EV71 5’ UTR, we developed RACE-Nm, a method that combines low-dNTP-based RACE with target enrichment sequencing (Fig 2A). This approach overcomes the limited sensitivity of conventional Nm-seq methods for low-abundance transcripts [5,34,35]. It enables direct, background-free identification of modification sites without the need for a control sample, offers high sensitivity suitable for low-abundance RNAs, and simplifies data analysis by directly mapping truncated cDNA ends. Using five designed primers, four primer extension products were successfully amplified and sequenced except Pr627 (Fig 2B). The specific poly-G-ended reads were aligned to the EV71 reference genome and visualized using IGV, where abrupt drops in read coverage (cliff-like patterns) indicated potential Nm modification sites (Fig 2C). Some such sites were identified, including 642A, which exhibited the highest modification rate and was detected by both primers Pr789 and Pr901 (Fig 2D and S2 Table). To establish biological relevance, we cross-referenced these experimentally identified sites with our NanoNm predictions from the EV71 XF strain by genome position conversion (Figs 1B and 2E). This integrated analysis identified three high-confidence Nm sites (307C, 528A, and 758C) that were consistently detected by both methods. The sites 307C and 528A are located within the IRES, whereas 758C resides in the CDS region. Notably, 307C and 528A are evolutionarily conserved across the enterovirus genus (Figs 2F and S2).

### FBL-catalyzed 2’-O-methylation of the EV71 genome inhibits viral protein synthesis

**Fig 3:**
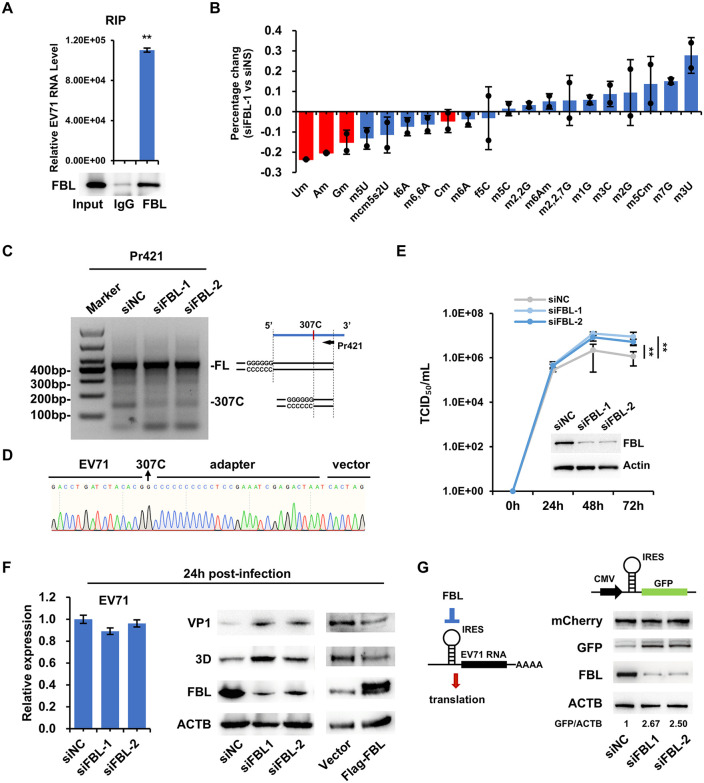
FBL mediates internal 2’-O-methylation of EV71 RNA. **(A)** RIP-qPCR analysis of FBL-EV71 RNA association in crosslinked RD cell lysates using anti-FBL antibody, while IgG served as negative control. **(B)** Relative abundance of Nm modifications in EV71 RNA from FBL-knockdown (siFBL-1) versus control cells by UHPLC-MS/MS. **(C)** Agarose gel analysis of PCR products from low-dNTP primer extension assays performed with or without FBL knockdown, showing reduced stalling at site 307C upon FBL depletion. **(D)** Representative Sanger sequencing chromatogram of TA clone confirming the RT termination event at position 307C. **(E)** FBL knockdown was performed in RD cells using two independent siRNAs, and the cells were then infected with EV71 at an MOI of 0.1 to permit multiple rounds of infection [56]. Viral growth kinetics (siFBL-1/2) versus control (siNC). Titers (TCID_50_/mL) were determined at indicated time points (n = 3 biological replicates). FBL knockdown efficiency was confirmed by western blot analysis. **(F)** FBL suppresses EV71 viral protein production. Left: qPCR analysis of EV71 RNA levels in RD cells with FBL-knockdown at 24 hpi (normalized to GAPDH). Middle/Right: Western blots of viral 3D and VP1 proteins under FBL knockdown or overexpression conditions. GAPDH served as loading control. **(G)** Proposed model: FBL-mediated suppression of IRES activity inhibits 3D protein synthesis. Western blot analysis indicated significantly enhanced GFP expression from the EV71 IRES reporter following FBL knockdown in 293T cells. GFP/actin ratios are shown below the image. An mCherry reporter was co-transfected as a transfection efficiency control. Data represent mean ± SD (n = 3 biological replicates); *p ≤ 0.05, **p ≤ 0.01 (two-tailed t-test).

Having established that the EV71 RNA genome contains Nm modifications, the responsible methyltransferase was subsequently investigated. RNA-seq analysis revealed that EV71 infection downregulates key 2’-O-methyltransferases, including FBL and FTSJ3 (S3A and S3B Fig, S3 Table), contrasting with the upregulation of m6A and m1A machineries [27,36]. To test their involvement, RIP assays were performed, which confirmed a specific interaction between FBL and EV71 RNAs that was not observed for FTSJ3 (Figs 3A and S3C). Accordingly, UHPLC-MS/MS analysis of virion RNAs from FBL-knockdown cells showed a substantial reduction in the Nm modifications (Fig 3B), confirming FBL as the primary enzyme depositing these internal methylations. Furthermore, FBL knockdown reduced the stalling signal of primer extension products at position 307 and 642 (Figs 3C and S3D). The stalling sites were confirmed by TA cloning (Figs 3D and S3E), demonstrating that FBL directly catalyzes 2′-O-methylation at sites 307C and 642A.

Given these findings, we hypothesized that FBL might play a role in EV71 replication. While FBL knockdown did not affect EV71 yield at 24 h post-infection (hpi), it significantly increased the virus productions at 48 and 72 hpi (Fig 3E), in contrast to the minimal effect of FTSJ3 knockdown (S3F Fig). Although FBL knockdown enhanced viral production only at later time points (48–72 hpi), it increased the expression of viral proteins (VP1 and 3D) without affecting viral RNA levels at 24 hpi (Fig 3F). Conversely, FBL overexpression suppressed viral protein production (Fig 3F). These results demonstrate that FBL-mediated restriction occurs primarily at the translational level. The delayed effect on viral production is likely the cumulative impact of this translational suppression on subsequent replication cycles, indicating that FBL targets IRES-mediated translation (Fig 3G). Using a reporter in which the EV71 IRES was fused to GFP [29], FBL knockdown significantly enhanced IRES-dependent GFP expression (Fig 3G). In contrast, FBL knockdown had no significant effect on the CrPV (Cricket paralysis virus) IRES-driven GFP expression, confirming the specificity of FBL-mediated regulation (S3G Fig). These results demonstrate that FBL suppresses viral infection by directly inhibiting the EV71 IRES, revealing its specific role in restricting cap-independent translation of EV71.

### Functional 307C Nm site represses IRES activity by attenuating PCBP2 binding

**Fig 4:**
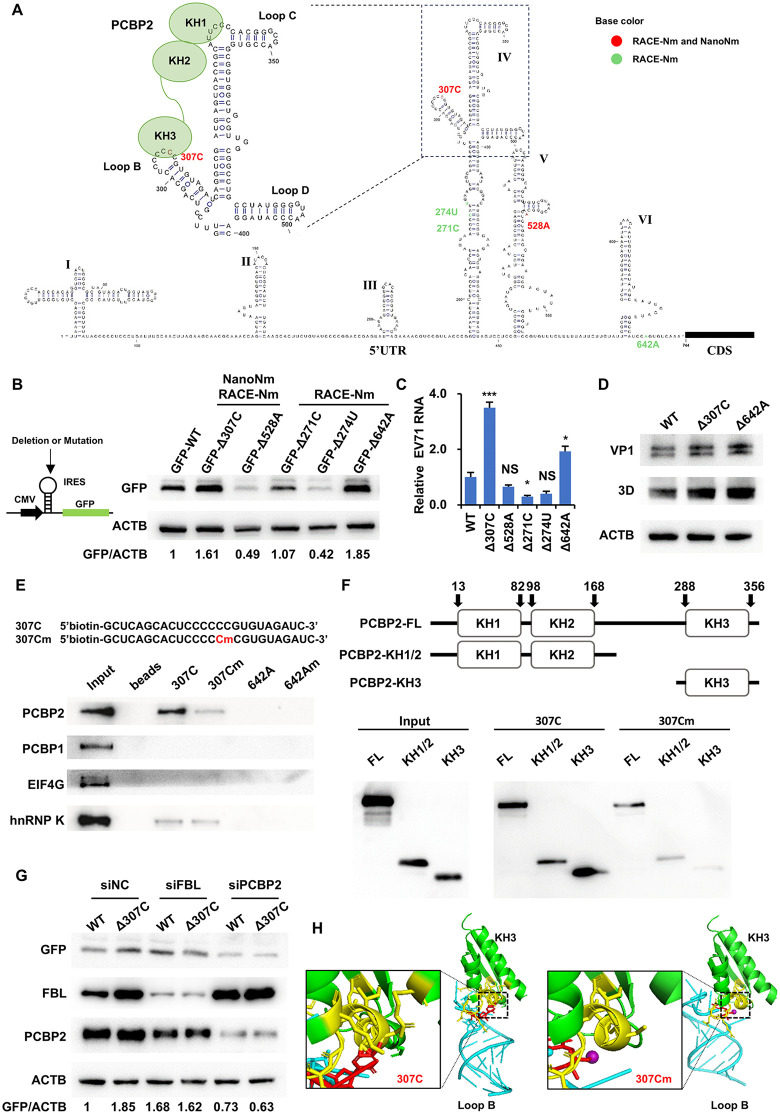
Functional analysis of Nm modification sites in the EV71 5′ UTR. **(A)** Secondary structure of the EV71 5′ UTR. Candidate Nm sites (271C, 274U, 307C, 528A, and 642A) are color-labelled by the identification method: red (detected by both RACE-Nm and NanoNm), or green (RACE-Nm-specific). Key functional domains including stem-loop IV and V are labeled. Interactions of PCBP2’s KH3 domain with loop B and KH1 domain with loop C are indicated. The IRES secondary structure was predicted using RNAfold (http://rna.tbi.univie.ac.at/cgi-bin/RNAWebSuite/RNAfold.cgi) and visualized by VARNA (https://varna.lisn.upsaclay.fr/). **(B)** Effect of Nm site deletion mutations on IRES-mediated translation. GFP expression from the EV71 IRES reporter construct (as in Fig 3G) was measured in cells transfected with wild-type or mutant reporters. GFP/actin ratios are shown below the image. **(C-D)** Viral RNA **(C)** and protein **(D)** levels of Nm site deletion mutants at 24 hours post-infection (MOI = 0.1) were evaluated by qPCR and Western blot, respectively. **(E)** RNA pulldown assay demonstrates that 307 Cm impairs PCBP2 binding. Biotin-labeled RNA oligonucleotides, either unmodified or containing a Cm at position 307, were used for the assays, with oligonucleotides containing 642A or 642Am serving as controls. Bound PCBP2 was detected by western blot. Binding of PCBP1, EIF4G, and hnRNPK were also assessed. **(F)** RNA pulldown assays were performed using the 307C/Cm oligonucleotides and lysates from cells expressing full-length PCBP2 (FL), the KH1-KH2 domains, or the KH3 domain. **(G)** GFP expression from the WT or mutant EV71 IRES reporters was measured in 293T cells under the indicated knockdown conditions (siPCBP2 and/or siFBL). GFP/actin ratios are shown below the image. **(H)** The interaction between the EV71 IRES loop and the PCBP2 KH3 domain was predicted using HelixFold3 (https://paddlehelix.baidu.com/app/all/helixfold3/forecast). Close-up views highlight the potential altered interaction introduced by the 2′-O-methyl group at C307 (red). Data represent mean ± SD (n = 3); *p ≤ 0.05, **p ≤ 0.01 (two-tailed t-test).

To determine the functional impact of specific Nm sites on IRES activity, five candidate sites within the EV71 IRES (271C, 274U, and 307C in stem-loop IV; 528A in stem-loop V; and 642A upstream of the coding region) were selected for mutagenesis based on their consistent detection or high RACE-Nm rates (Fig 4A). Deletion mutations were introduced into the EV71 IRES reporter construct (as used in Fig 3G). While most mutations markedly reduced IRES-driven GFP expression, deletion of 307C and 642A enhanced translation (Fig 4B). In contrast, point mutations (A, U, or G) did not increase GFP expression (S4A Fig), indicating that the identity of the base, not merely the loss of sequence, influences IRES function. To assess the effects on viral replication, the same deletions were introduced into infectious EV71 clone. Although most deletions severely impaired virus production, the Δ307C mutant exhibited a growth curve and virion yield similar to those of the WT (S4B Fig). Strikingly, infection with Δ307C and Δ642A mutants at an MOI of 0.1 resulted in significantly elevated viral RNA (Fig 4C) and protein (Fig 4D) levels at 24 hours post-infection. The enhanced translation indicated a critical role of 2’-O-methylation in regulation of the viral lifecycle.

The sequence context of the 307C site is located within a conserved poly-C tract in loop B of stem-loop IV (Fig 4A). Its 2′-O-methylation might interfere with the binding of Poly(rC) Binding Protein 2 (PCBP2) [29,37,38], which is known to recognize poly-C motifs via its KH domains [39,40]. The Cryo-EM structures reveal that its KH3 domain primarily binds loop B, while KH1 binds loop C, collaboratively compacting the RNA into a stable, globular complex essential for enterovirus translation (Fig 4A) [38]. To test this, RNA pulldown assays were performed using oligonucleotides containing either an unmodified C or a Cm at position 307, with oligonucleotides containing the 642A or 642Am sites used as controls. The Cm modification reduced PCBP2 binding, whereas binding of PCBP1 [41] and EIF4G [22] was undetectable, and hnRNPK [42] binding was unaffected by the modification (Fig 4E). In contrast, no significant binding of PCBP1, PCBP2, EIF4G, or hnRNPK was detected with either the 642A or 642Am probe (Fig 4E). To further confirm the altered binding by 307 Cm, EMSA assays were performed with gradient concentrations of purified Flag-PCBP2 protein. As shown in S4C Fig, the 307 Cm modification consistently reduced PCBP2 binding across all protein concentrations tested, supporting the inhibitory effect. Subsequent pulldown assays with truncated PCBP2 confirmed that this poly-C motif interacts with both KH1/2 and KH3 domains *in vitro* and that the interactions are decreased by 307 Cm (Fig 4F). Knockdown of PCBP2 substantially impaired IRES activity, and the effect was partially rescued by concurrent depletion of FBL (S4D Fig). Furthermore, the translational enhancement normally conferred by the Nm modification in the Δ307C mutant was abolished upon knockdown of either FBL or PCBP2 (Fig 4G). The comparative analysis of the binding interfaces between the KH3 domain and loop B using HelixFold3 [43] revealed that the predicted structure closely resembles the crystal structure of the KH3 domain (PDB: 2P2R) [44], with 307C located within the loop region. However, Nm modification at this site creates a distinct interaction environment. Specifically, 307 Cm induces local conformational rearrangements in the RNA backbone and base orientation, leading to an altered interaction network with the PCBP2 KH3 domain, as evidenced by reduced proximal residues (from 14 to 11 within 5 Å) and a distinct binding interface (Fig 4H). Together, these findings establish that FBL-mediated 2′-O-methylation at C307 represses IRES-dependent translation by specifically inhibiting the recruitment of PCBP2.

### FBL suppresses EV71 replication through a 3D-dependent host gene regulatory axis

**Fig 5:**
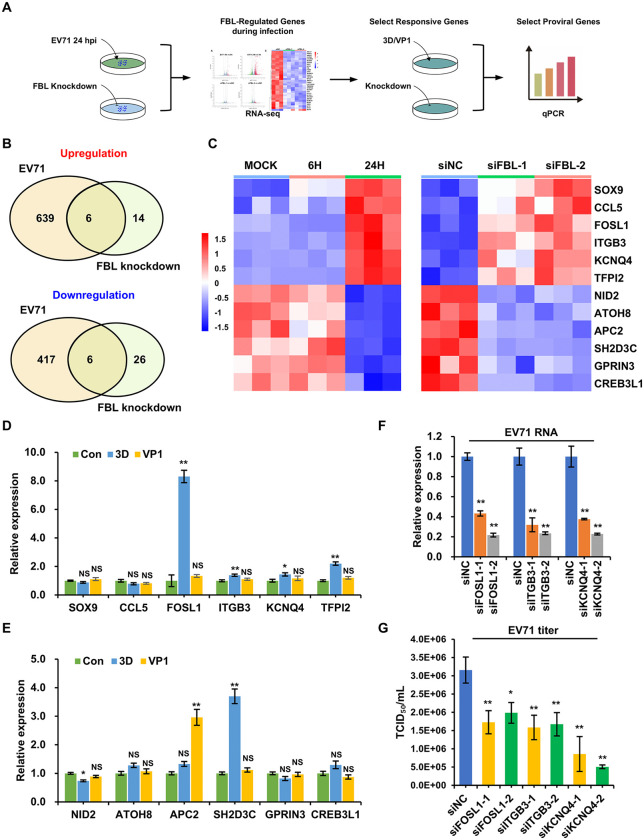
FBL suppresses EV71 replication through host gene regulation. **(A)** A workflow for identifying and validating FBL-regulated host genes critical for EV71 replication. **(B)** Venn diagram of overlapping differentially expressed genes between FBL knockdown (both regulated by siFBL-1/2) and EV71 infection (24 hpi). **(C)** Heatmap displaying expression of the 12 candidate genes (6 upregulated, 6 downregulated) that are consistently modulated under both FBL-knockdown and EV71 infection conditions (24 hpi). **(D-E)** qPCR analysis of host gene expression in 293T cells overexpressing EV71 3D or VP1, showing regulation of candidate genes identified in Fig 5C (normalized to GAPDH). (F-G) knockdown of FOSL1, ITGB3, or KCNQ4 were performed in RD cells with two siRNAs for each gene, and the cells were infected with EV71 (MOI = 0.1). EV71 RNA levels following knockdown of pro-viral host genes (FOSL1, ITGB3, KCNQ4) were examined via qPCR at 24 hpi **(F)**. Viral titers (TCID₅₀/mL) in the supernatant were determined under the same conditions **(G)**. Data in D-G represent mean ± SD (n = 3 biological replicates); *p ≤ 0.05, **p ≤ 0.01 (two-tailed t-test).

To determine if FBL-mediated translational repression influences the host transcriptome to restrict EV71, a screening workflow was designed to identify crucial FBL-regulated host genes during infection (Fig 5A). We profiled differentially expressed genes (DEGs) in cells transfected with two independent FBL-targeting siRNAs followed by EV71 infection, identifying 52 DEGs (20 upregulated, 32 downregulated) upon FBL depletion (S5A, S5B and S5C Figs, S4 Table). Cross-referencing these with transcriptional changes from EV71 infection at 24 hpi revealed a core set of 12 consistently modulated candidate genes (6 upregulated, 6 downregulated; Figs 5B and 5C). Their regulation was confirmed by qPCR under both FBL-knockdown and infection conditions (S5D and S5E Figs). A third FBL-targeting siRNA (siFBL-3), along with an additional negative siRNA control, was tested to further rule out off-target effects. While the first two siRNAs consistently regulated all 12 candidate genes, the third siRNA showed inconsistent regulation for CCL5, TFPI2, and CREB3L1, indicating these are off-target hits. These host gene changes might be an indirect consequence of FBL’s suppression of viral protein production. To test this, the viral proteins VP1 or 3D were individually expressed in uninfected cells, followed by analysis of the expressions of 12 candidate genes (Figs 5D and 5E). Strikingly, 3D expression significantly upregulated four genes, including FOSL1, ITGB3, KCNQ4, and TFPI2, whereas VP1 had no expected effect. Given that TFPI2 was inconsistently regulated by three FBL siRNAs, we focused on FOSL1, ITGB3, and KCNQ4 for further validation.

Notably, FOSL1, a critical component of the AP-1 transcription factor complex, exhibited a dramatic ~10-fold increase. This indicates that FBL, by limiting 3D protein synthesis, indirectly prevents the aberrant activation of these specific host factors. Furthermore, depletion of FOSL1, ITGB3, or KCNQ4 significantly impaired viral RNA expression and infectious particle production (S5F Fig, Fig 5F and 5G), suggesting their roles as crucial pro-viral factors. Collectively, the above data establish a novel antiviral pathway in which FBL suppresses EV71 replication through a regulatory cascade: FBL-mediated 2’-O-methylation inhibits IRES-dependent translation, which limits the accumulation of the 3D protein, thereby preventing the subsequent activation of pro-viral host genes and ultimately restricting infection.

### Deletion of 307C or 642A enhances viral replication and pathogenicity in mice

**Fig 6:**
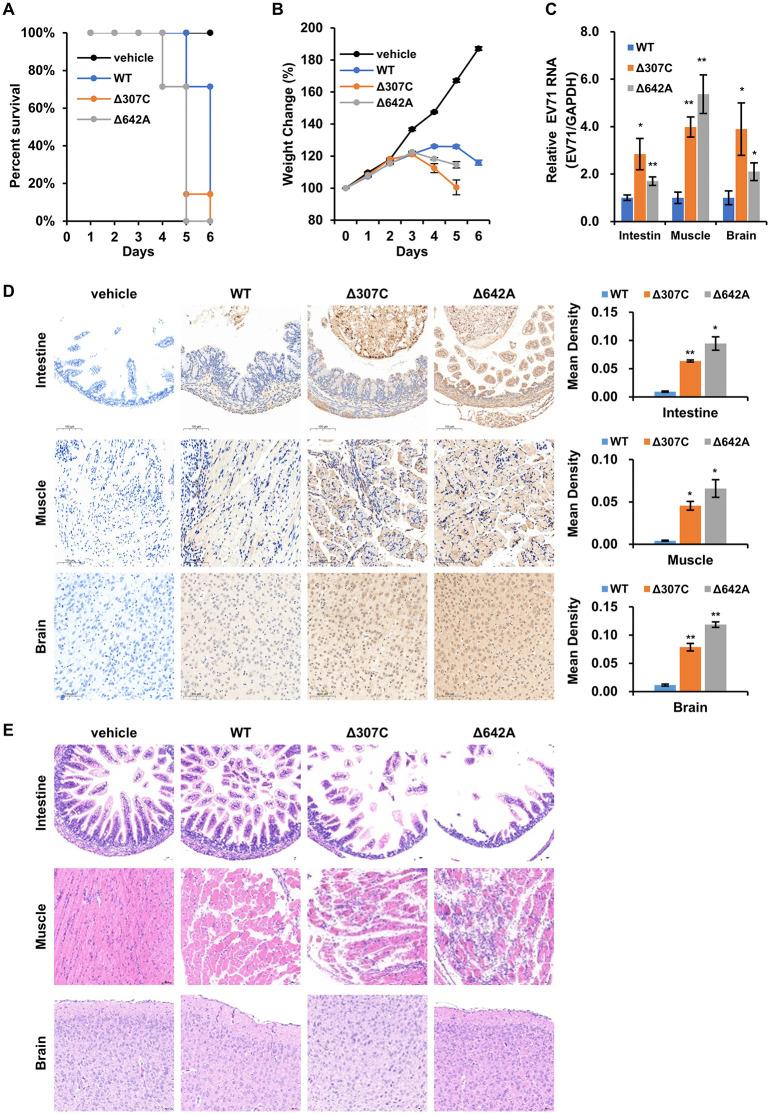
Deletion of 307 Cm or 642Am enhances EV71 replication and pathogenicity in mice. **(A)** Survival curves of AG6 mice infected with EV71 wild-type (WT), Δ307C, or Δ642A virus (n = 7 per group). **(B)** Average body weight change of infected mice over time. **(C)** Viral RNA loads in muscle, intestine and brain at 3 days post-infection, as measured by qRT-PCR. **(D)** Immunohistochemical detection of VP1 in muscle, intestine, and brain sections. Increased VP1 signal indicates higher viral protein levels in mice infected with Δ307C or Δ642A mutants. The bar graphs on the right quantified the VP1 expression. **(E)** Histopathological analysis of limb muscle, brain, and intestine. Representative images show tissue damage across all organs examined: limb muscle exhibits myofiber degeneration and inflammatory cell infiltration; the brain shows signs of neuronal injury; and the intestine is characterized by villous atrophy, crypt loss, and inflammatory cell infiltration. Data represent mean ± SD (n = 7); *p ≤ 0.05, **p ≤ 0.01 (two-tailed t-test).

To assess the pathogenic impact of specific Nm modifications, we infected AG6 interferon receptor-deficient mice with EV71 wild-type, Δ307C, or Δ642A mutant viruses. Mice infected with either mutant exhibited shorter survival times with mortality kinetics accelerated by 1–2 days compared to the WT control, although all infections were ultimately fatal (Fig 6A). Concurrently, these mice showed more rapid weight loss compared to the WT control (Fig 6B). This accelerated disease progression correlated with markedly higher viral RNA loads in all tissues examined, including muscle, intestine, and brain (Fig 6C). Notably, in muscle tissue, viral RNA levels in mutant-infected mice were more than 4-fold higher than in WT controls (Fig 6C). Immunohistochemistry (IHC) confirmed enhanced pathogenicity at the protein level, revealing substantially stronger and more widespread VP1 staining in tissues infected with the Δ307C or Δ642A mutants. This pattern was consistently observed across all tissues and correlated closely with the elevated viral RNA loads, indicating robust viral protein synthesis and dissemination (Fig 6D). Histopathological analysis provided direct evidence of the functional consequences of this enhanced replication, revealing aggravated tissue damage across all organs. In limb muscle, mutant virus infection resulted in severe myofiber degeneration, accompanied by dense inflammatory infiltration. The brain sections from mutant-infected mice displayed pronounced neuronal injury, indicating severe neuropathology. The most dramatic pathology was observed in the intestine, which exhibited severe mucosal damage characterized by villous atrophy, crypt loss, and immune cell infiltration (Fig 6E). The overall histopathological scores for all organs were significantly higher in mice infected with the Nm-deficient mutants. However, the double mutant (Δ307C/Δ642A) did not exhibit statistically significant additive effects compared to the Δ307C single mutant (S6 Fig), indicating that the two Nm sites act independently. Collectively, these data demonstrate that the loss of 2′-O-methylation at sites 307 and 642 enhances EV71 replication and markedly exacerbates viral pathogenicity in vivo.

## Discussion

**Fig 7:**
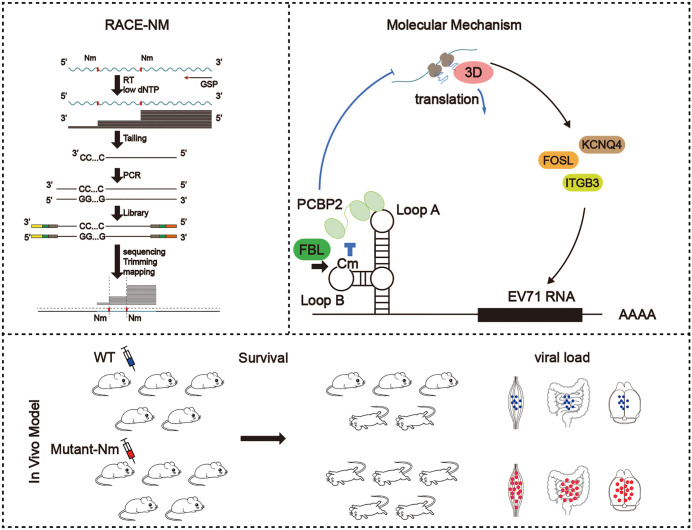
Graphic abstract. Left top: Establishment of RACE-Nm, a sensitive sequencing method for mapping internal Nm modifications on low-abundance viral RNAs at single-nucleotide resolution. Right top: Mechanism of FBL-mediated translational repression. The methyltransferase FBL installs an Nm modification at position 307C within the EV71 IRES. This 2’-O-methylation creates steric hindrance that blocks PCBP2 recruitment to the IRES, thereby repressing cap-independent translation. Bottom: FBL-mediated Nm modification suppresses viral pathogenesis.

Our study identifies a novel antiviral mechanism that the host methyltransferase FBL installs internal 2’-O-methylation (Nm) on the EV71 RNA genome to restrict viral replication and pathogenesis (Fig 7). The application of a newly developed, sensitive RACE-Nm method enabled the identification of a critical Nm site at 307C within the IRES (Fig 7, left). Methylation at this site by FBL disrupts PCBP2 binding, leading to the inhibition of IRES-dependent translation and a consequent reduction in the synthesis of the viral 3D polymerase (Fig 7, right). This cascade ultimately prevents the subsequent activation of pro-viral host genes (Fig 7, right). The physiological relevance of this FBL-mediated restriction was confirmed in vivo, as loss of the Nm modification resulted in enhanced viral pathogenicity (Fig 7, bottom). Collectively, our findings establish internal Nm as a crucial epitranscriptomic regulator of host-virus interactions.

This inhibitory function of internal Nm in an uncapped RNA virus represents a paradigm distinct from its established role in capped viral RNAs. Unlike in coronaviruses or influenza viruses, where Nm modifications are strategically confined to the 5’ cap to evade innate immune sensing [15,16,45], EV71, lacking a canonical cap, leverages internal Nm sites to directly fine-tune its replication cycle. This suggests that the Nm ‘signature’ of a virus is not defined by its cap status alone, but rather by a complex interplay between its replication strategy and the host’s RNA modification machinery [12,46]. Our work, alongside the discovery of FTSJ3-mediated internal Nm in HIV [17], establishes internal Nm methylation as a universal regulatory strategy in different viral families.

RACE-Nm is a high-sensitivity method for mapping internal Nm modifications. A central finding of our study is the mechanistic elucidation of how a single Nm modification at site 307C can exert a profound inhibitory effect on viral translation. This discovery was enabled by our development of RACE-Nm, a method that combines low-dNTP–based reverse transcription with target enrichment sequencing to map internal Nm sites at single-nucleotide resolution. Critically, unlike 2′OMe-seq [35], Nm-seq [5], and Nm-Mut-seq [34], which rely on a chemical treatment step and require a matched, unmodified “input” sample for background subtraction, our RACE-Nm method captures Nm sites through specific reverse transcription termination and subsequent poly-G tailing. This generates uniquely tagged cDNA molecules that allow for direct, background-free identification of modification sites without the need for an input control. Furthermore, the subsequent PCR amplification and target-enrichment steps make RACE-Nm particularly suited for profiling low-abundance RNAs, such as viral transcripts, with high sensitivity from limited sample input. The data analysis is also more straightforward, as it involves simply mapping the truncated cDNA ends to the reference genome, bypassing the complex normalization and background subtraction required by other methods.

FBL orchestrates a multi-layered antiviral strategy by suppressing viral IRES activity and blocking the subsequent reprogramming of the host transcriptome. Prior work from our group and others has demonstrated the pro-viral roles of various epitranscriptomic marks, including m6A, m5C, and ac4C, in facilitating virus RNA replication [27–29,47]. The inhibitory role of the Nm modification in the current study provides a clear functional contrast, revealing it as a host-derived antiviral mechanism. Mechanistically, methylation of C307 within the conserved poly-C tract of stem-loop IV impedes the binding of PCBP2, a critical host factor that promotes both genome replication [48] and IRES assembly [29,37,38,49]. This represents a novel mechanism of translational control where an epitranscriptomic mark directly modulates RNA-protein interactions crucial for cap-independent translation. Thus, 2′-O-methylation at C307 acts not merely as a passive mark but as a precise steric hindrance switch that disrupts PCBP2 binding and represses IRES-dependent translation [50]. Nevertheless, FBL knockdown enhances EV71 translation through multiple mechanisms. Beyond the C307 site, loss of methylation at A642 also promotes IRES activity (Fig 4B). However, the mechanism underlying this translational repression remains unknown. Pulldown assays ruled out binding to PCBP1, PCBP2, EIF4G, and hnRNPK (Fig 4E), suggesting that A642 acts through a distinct mechanism. In addition to the 5’UTR, NanoNm analysis identified numerous Nm sites within the EV71 coding region. Given that Nm within coding regions can impair translation elongation in cellular mRNAs [6], it is plausible that similar mechanisms operate on the viral genome. Future studies are needed to explore this possibility. Therefore, the translational enhancement upon FBL depletion may reflect the cumulative loss of Nm marks across the viral genome, not solely the 307C site.

By suppressing the IRES, FBL limits the synthesis of the viral 3D polymerase, which in turn indirectly prevents the upregulation of key pro-viral host genes, including FOSL1, ITGB3, and KCNQ4. This effectively compromises a host environment that is primed to support robust viral replication [51]. This indirect mechanism explains the delayed yet potent antiviral phenotype observed upon FBL knockdown, where enhanced translation at early timepoints culminates in amplified replication cycles later in infection. This finding also presents an interesting contrast to the mechanism previously reported, where EV71 infection was shown to upregulate the transcription factor AP1, which in turn drives the expression of miR-146a to suppress type I interferon production and facilitate immune evasion [52]. Our work suggests the host can counter this by employing FBL to restrict the very synthesis of viral proteins, including 3D, which is necessary to activate FOSL1 (a component of AP1 complex). This suggests a complex interplay where the virus manipulates host transcription factors for immune suppression, and the host, in turn, utilizes RNA modification to suppress viral translation, thereby blocking subsequent viral manipulation of the host transcriptome.

RNA modifications function as critical yet divergent regulators of viral pathogenesis. While certain modifications, such as m6A, m5C, and ac4C, generally enhance viral replication and pathogenicity by stabilizing viral RNA, promoting translation, or aiding immune evasion [27–29], the present work establishes that FBL-mediated 2’-O-methylation constrains EV71 pathogenesis, revealing a distinct antiviral mechanism. This unique role is underscored by the markedly enhanced viral pathogenicity in mice upon deletion of the Nm sites 307C or 642A. The mechanistic basis of this regulation is not attributable to sequence loss or structural disruption, as the Δ307C mutation resides in a loop region and is not predicted to perturb local RNA secondary structure. Rather, the loss of the repressive Nm mark itself relieves IRES suppression, thereby exacerbating disease. Furthermore, the observation that point mutations (A/U/G) at this site fail to enhance IRES activity confirms the essential role of cytosine identity. We propose that nucleotide substitution alters the local sequence context, potentially disrupting FBL recruitment or the assembly of a functional RNA-protein complex, ultimately resulting in constitutively low translation efficiency independent of methylation.

Our work defines a complete antiviral pathway mediated by FBL-dependent RNA 2’-O-methylation, from target recognition to translational suppression and in vivo pathogenesis. The development of the RACE-Nm method enables sensitive profiling of internal Nm modifications in low-abundance viral RNAs. Despite these advances, several intriguing questions remain. First, it is worth noting that FBL-mediated attenuation of viral infection may result not only from inhibition of 3D-dependent upregulation of pro-viral host genes but also from suppression of viral protein synthesis. A key subsequent question is how 3D upregulates FOSL1, ITGB3, and KCNQ4, and what their functional roles are in the viral life cycle. Given the around 10-fold induction of FOSL1 upon 3D expression, transcriptional activation is proposed. Subsequently, FOSL1 may promote other host factors for viral replication, while ITGB3 could facilitate viral entry or signaling. Although our data suggest that increased 3D synthesis from the Δ307C mutant may drive this upregulation, direct causality requires future study. In addition, we cannot exclude the possibility that other viral non-structural proteins may also contribute to the upregulation of FOSL1, ITGB3, and KCNQ4. Systematic testing of all non-structural proteins would be required to establish the specificity of 3D in this process. Second, what is the complete repertoire of viral and host factors that recognize internal Nm modifications and mediate their effects? Are there dedicated ‘readers’ beyond the inferred disruption of PCBP2 binding, perhaps like YTH domain proteins that read m6A marks [53,54]? Third, how is FBL specifically recruited to the EV71 RNA genome? The discovery of guiding snoRNAs and conserved motifs warrants further investigation, as snoRNA-mediated targeting is a hallmark of FBL’s function in ribosomal RNA methylation [55]. Finally, the therapeutic implications are substantial. Could small-molecule activators of FBL or oligonucleotides designed to mask specific Nm sites be developed as broad-spectrum antivirals against enteroviruses and other picornaviruses?

In conclusion, our study redefines the role of internal 2’-O-methylation from a fundamental RNA modification to a potent antiviral defense mechanism. We have delineated a comprehensive pathway from the writer (FBL) and the mark (internal Nm) to the molecular consequence (IRES inhibition) and the physiological outcome (attenuated pathogenesis). This work sheds new sights on antiviral research, targeting the epitranscriptomic landscape could yield innovative strategies to combat viral infections.

## Supporting information

S1 Fig Validation of low-dNTP primer extension method for detecting Nm modifications. (A) Overlap of Nm sites (methylation rate ≥ 0.1) identified across triplicate virus infected samples by NanoNm. (B) The assay was firstly validated using a 100-nt synthetic HIV RNA probe (P-2’O, 733–832 nt) containing known Am783 (position 51) and artificial Cm (position 21) sites. (C) Schematic workflow, including low-dNTP reverse transcription, template-independent poly(C) tailing, PCR amplification with adapter primers, and TA cloning for sequencing validation. (D) FAM fluorescence visualization of RT termination products at modified positions. (E) Agarose gel electrophoresis of PCR-amplified termination product. Sanger sequencing chromatogram confirming RT stalling at HIV Am783 through TA cloning of 5’ RACE products.
(TIF)

S2 Fig Evolutionary conservation analysis of identified Nm sites across multiple enterovirus species. (TIF)

S3 Fig FBL is the primary methyltransferase for EV71 RNA. (A) RNA-seq was performed with total RNA extracted from EV71-infected (MOI = 0.1) RD cells at 0, 6, and 24 hours post EV71 infection. The heatmap illustrates the expression levels of genes associated with m6A, m1A, and Nm modifications in RD cells. Rows: individual genes; columns: time points. Color scale represents Z-scores of FPKM values. (B) Western blot analysis of FBL and viral VP1 protein levels during infection (6 and 24 hpi). Actin serves as loading control. Relative FBL protein expression normalized to actin is indicated below the gel. (C) RIP-qPCR analysis of FTSJ3 binding to EV71 RNA. Crosslinked lysates from EV71-infected RD cells were immunoprecipitated with anti-FTSJ3 antibody or control IgG. Associated EV71 RNA levels were quantified by qPCR and normalized to input. (D) Agarose gel analysis of PCR products derived from low-dNTP primer extension assays using two independent primers, showing reduced reverse transcription stalling at site 642A under FBL knockdown conditions. (E) Representative Sanger sequencing chromatogram confirming the reverse transcription termination event at position 642A. (F) FTSJ3 knockdown has minimal effect on EV71 replication. Viral growth kinetics in RD cells (MOI = 0.1) transfected with control siRNA (siNC) or FTSJ3-targeting siRNA (siFTSJ3–1). Viral titers in the supernatant were determined by TCID₅₀ assay at the indicated time points. Knockdown efficiency was confirmed by Western blot. (G) The CrPV IRES (Cricket paralysis virus, AF218039.1, IRES 302–708) was inserted between the CMV promoter and the GFP gene, with the same backbone as the EV71 IRES reporter [29]. FBL knockdown did not significantly alter GFP expression from this reporter. An mCherry reporter was co-transfected as a transfection control.
(TIF)

S4 Fig Characterization of Nm site mutations. (A) Effect of nucleotide substitution at site 307 on IRES activity. GFP expression was assessed after introducing point mutations (A, U, or G) at position 307. GFP/actin ratios are shown below the image. (B) Viral production of EV71 Nm deletion mutants. Supernatants from cells transfected with wild-type or mutant infectious RNAs were harvested and titrated. (C) EMSA analysis of PCBP2 binding to FAM-labeled RNA probes containing either unmodified 307C or 2’-O-methylated 307 Cm. Flag-tagged PCBP2 was overexpressed in 293T cells and eluted from anti-Flag beads using 3 × Flag peptide. The eluted protein was quantified and used at three gradient concentrations in the binding reactions. The 307 Cm modification consistently reduced PCBP2 complex formation compared to the unmodified 307C probe across all concentrations tested. Free probes and PCBP2-RNA complexes are indicated. The right panel shows the quantification of bound RNA calculated as the percentage of shifted RNA to free probes. (D) GFP expression from the EV71 IRES reporter was measured in 293T cells under the indicated knockdown conditions (siPCBP2 and/or siFBL). GFP/actin ratios are shown below the image.
(TIF)

S5 Fig Identification of FBL-regulated host genes. (A) Volcano plots showing differentially expressed genes (DEGs) under four conditions: EV71 infection at 6 hpi, EV71 infection at 24 hpi, siFBL-1 transfection, and siFBL-2 transfection. (B) Venn diagrams depicting the overlap of upregulated (left) and downregulated (right) differentially expressed genes (DEGs) between siFBL-1 and siFBL-2 transfected cells, using thresholds of |log^2^FC| > 0.5 and Q-value < 0.01. (C) Heatmap of the 20 upregulated and 32 downregulated consensus DEGs identified in FBL-knockdown cells (from B). Rows represent genes; columns represent samples. (D-E) qPCR validation of the upregulated or downregulated host genes from panel Fig 5B and 5C. Host gene expression changes upon EV71 infection (D). Host gene expression changes upon FBL knockdown (E). A third FBL-targeting siRNA (siFBL-3), along with an additional negative siRNA control, was tested. GAPDH was used as internal normalization control. (F) Knockdown of FOSL1, ITGB3, or KCNQ4 were performed in RD cells with two siRNAs for each gene, and the cells were infected with EV71 (MOI = 0.1). The knockdown efficiency was verified via qPCR. Data show mean ± SD (n>=3 biological replicates); *p ≤ 0.05, **p ≤ 0.01 by two-tailed Student’s t-test.
(TIF)

S6 Fig Comparison of pathogenicity between Δ307C and Δ307C/Δ642A double mutant. (A) Survival curves of AG6 mice infected with EV71 Δ307C or Δ307C/Δ642A double mutant virus (n = 7 per group). (B) Average body weight change of infected mice over time. (C) Viral RNA loads in muscle, intestine, and brain at 3 days post-infection, as measured by qRT-PCR (n = 6 per group).
(TIF)

S1 Table List of high-confidence Nm sites identified by NanoNm in EV71 RNA. (XLSX)

S2 Table List of Nm sites identified by RACE-Nm in the EV71 5’UTR. (XLSX)

S3 Table RNA-seq analysis of differentially expressed genes upon EV71 infection. (XLSX)

S4 Table RNA-seq analysis of differentially expressed genes upon FBL knockdown. (XLSX)

S5 Table Oligo information. (XLSX)

S1 Raw Gel Compiled and annotated raw blot/gel images (S1 Raw images.pptx) and the corresponding unprocessed source files (S1 Raw images source.zip). The source files contain the original, uncropped images for all blots/gels presented in the figures.
(PPTX)
