# Supplementary material for: Fibrillarin-mediated 2’-O-methylation serves as a translation brake on uncapped enterovirus RNA
Source: PLoS Pathog. 2026 Jul 20;22(7):e1014455. doi: 10.1371/journal.ppat.1014455 (PMC13405287; doi:10.1371/journal.ppat.1014455)
Supplement: S1 Raw Gel — The source files contain the original, uncropped images for all blots/gels presented in the figures. (PPTX) [file ppat.1014455.s012.pptx]

## Slide 1
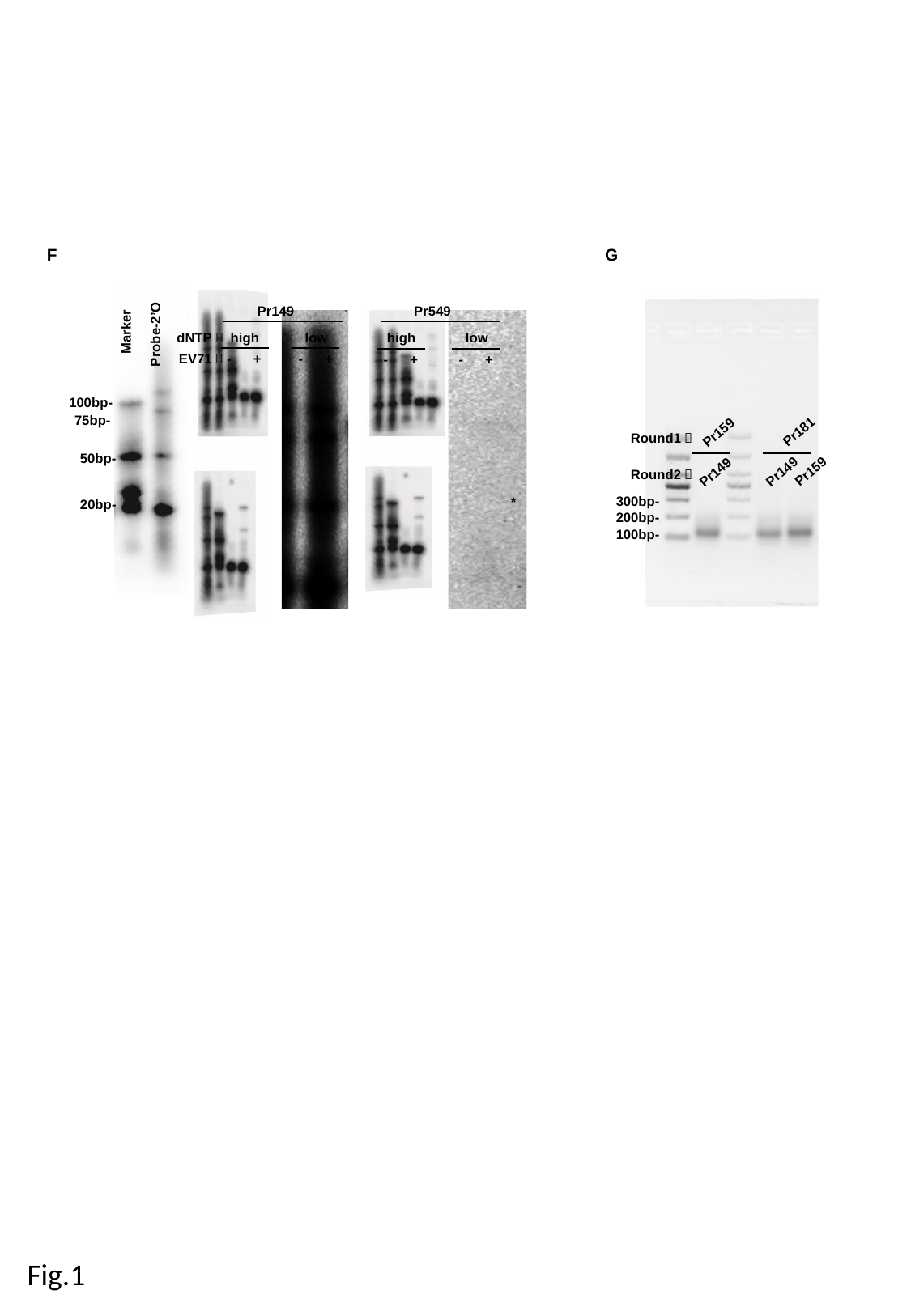

F
G
Pr549
Pr149
Marker
Probe-2’O
dNTP：
high
low
high
low
EV71：
-
+
-
+
-
+
-
+
100bp-
75bp-
Pr181
Pr159
Round1：
50bp-
Pr159
Pr149
Pr149
Round2：
300bp-
*
20bp-
200bp-
100bp-
Fig.1

## Slide 2
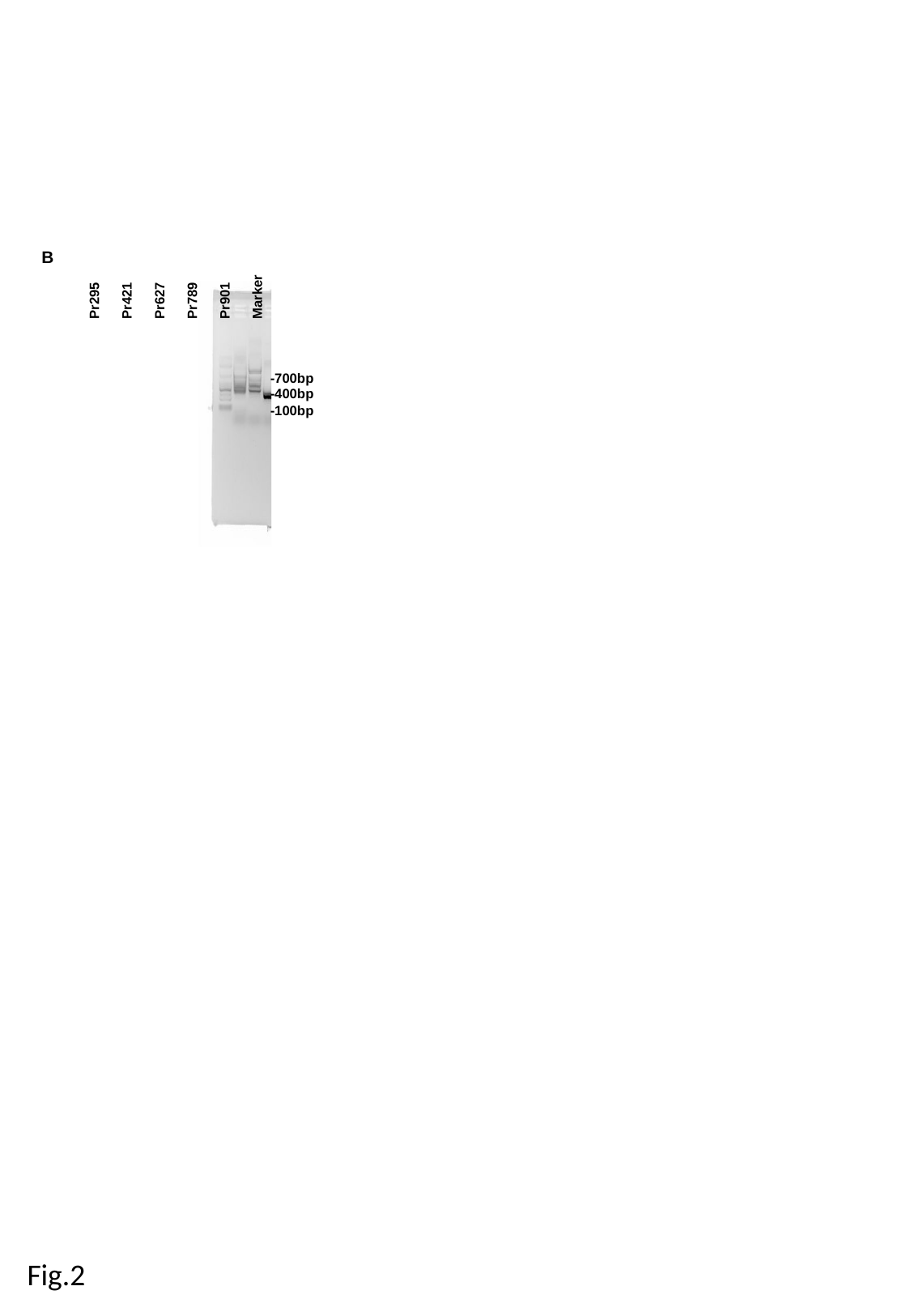

B
Pr627
Pr789
Pr901
Marker
Pr295
Pr421
-700bp
-400bp
-100bp
Fig.2

## Slide 3
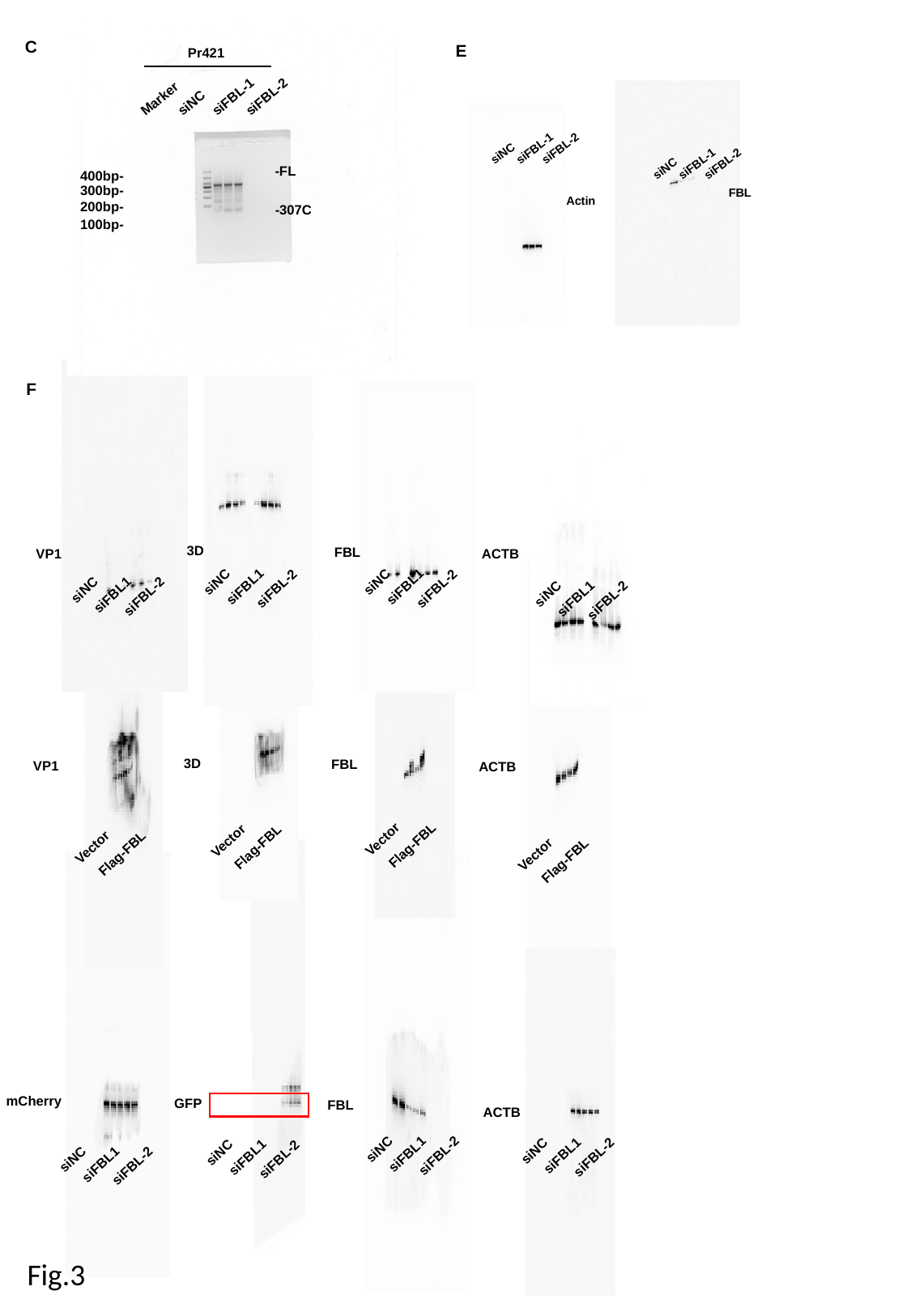

C
E
Pr421
siFBL-1
siFBL-2
Marker
siNC
siNC
siFBL-1
siFBL-2
siNC
siFBL-1
siFBL-2
-FL
400bp-
300bp-
FBL
Actin
200bp-
-307C
100bp-
F
3D
FBL
VP1
ACTB
siNC
siFBL1
siFBL-2
siNC
siFBL1
siFBL-2
siNC
siFBL1
siFBL-2
siNC
siFBL1
siFBL-2
3D
FBL
VP1
ACTB
Vector
Vector
Vector
Flag-FBL
Flag-FBL
Vector
Flag-FBL
Flag-FBL
mCherry
GFP
FBL
ACTB
siNC
siFBL1
siFBL-2
siNC
siFBL1
siFBL-2
siNC
siFBL1
siFBL-2
siNC
siFBL1
siFBL-2
Fig.3

## Slide 4
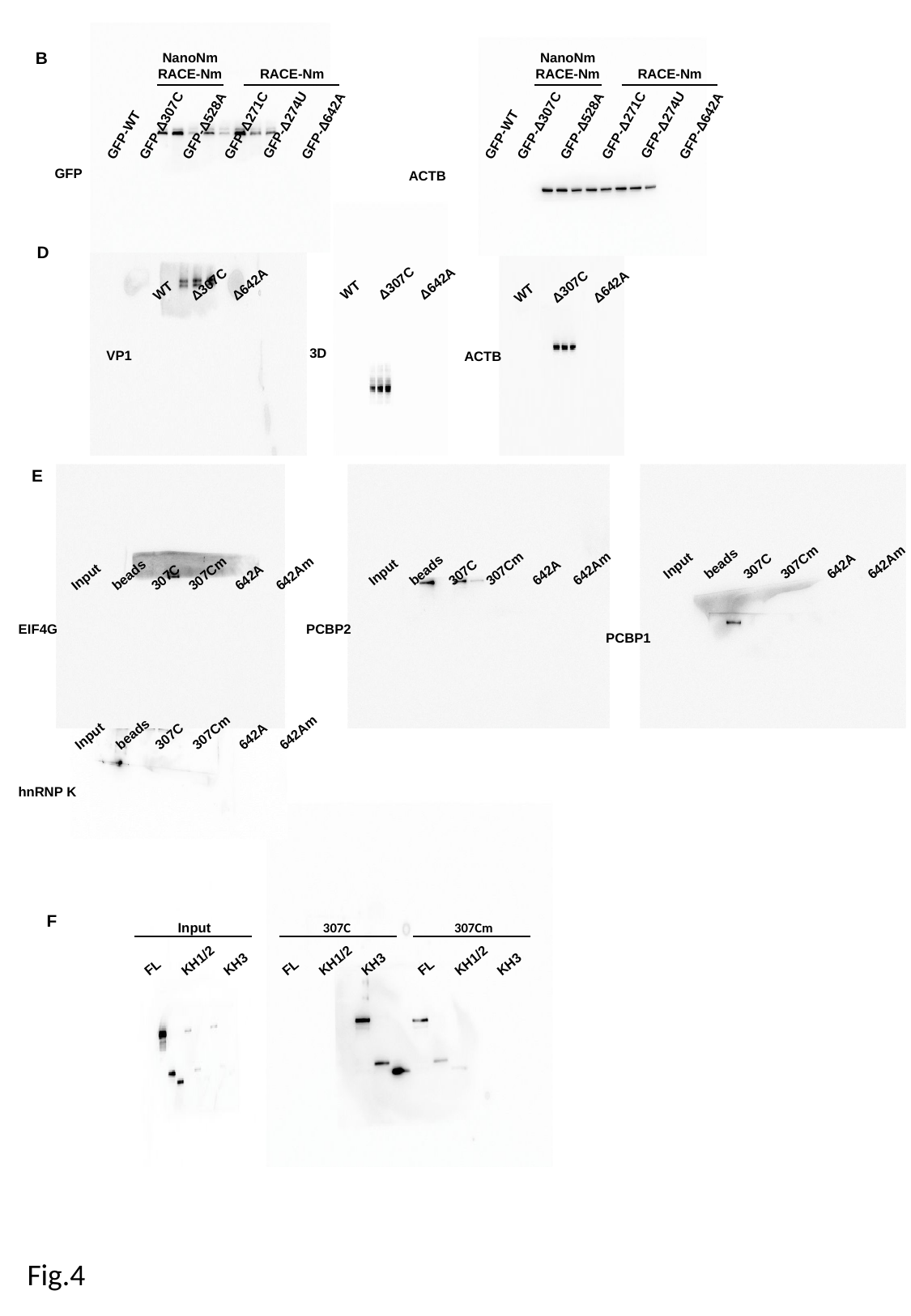

B
NanoNm
RACE-Nm
NanoNm
RACE-Nm
RACE-Nm
RACE-Nm
GFP-Δ528A
GFP-Δ528A
GFP-Δ307C
GFP-Δ307C
GFP-Δ271C
GFP-Δ271C
GFP-Δ274U
GFP-Δ274U
GFP-Δ642A
GFP-Δ642A
GFP-WT
GFP-WT
Δ307C
Δ307C
Δ642A
Δ642A
Δ307C
Δ642A
WT
WT
WT
3D
VP1
ACTB
GFP
ACTB
D
E
307Cm
642Am
307C
642A
307Cm
642Am
beads
Input
307C
642A
307Cm
642Am
307C
642A
beads
Input
beads
Input
EIF4G
PCBP2
PCBP1
307Cm
642Am
307C
642A
beads
Input
hnRNP K
F
Input
307C
307Cm
KH3
KH1/2
FL
KH3
KH1/2
FL
KH3
KH1/2
FL
Fig.4

## Slide 5
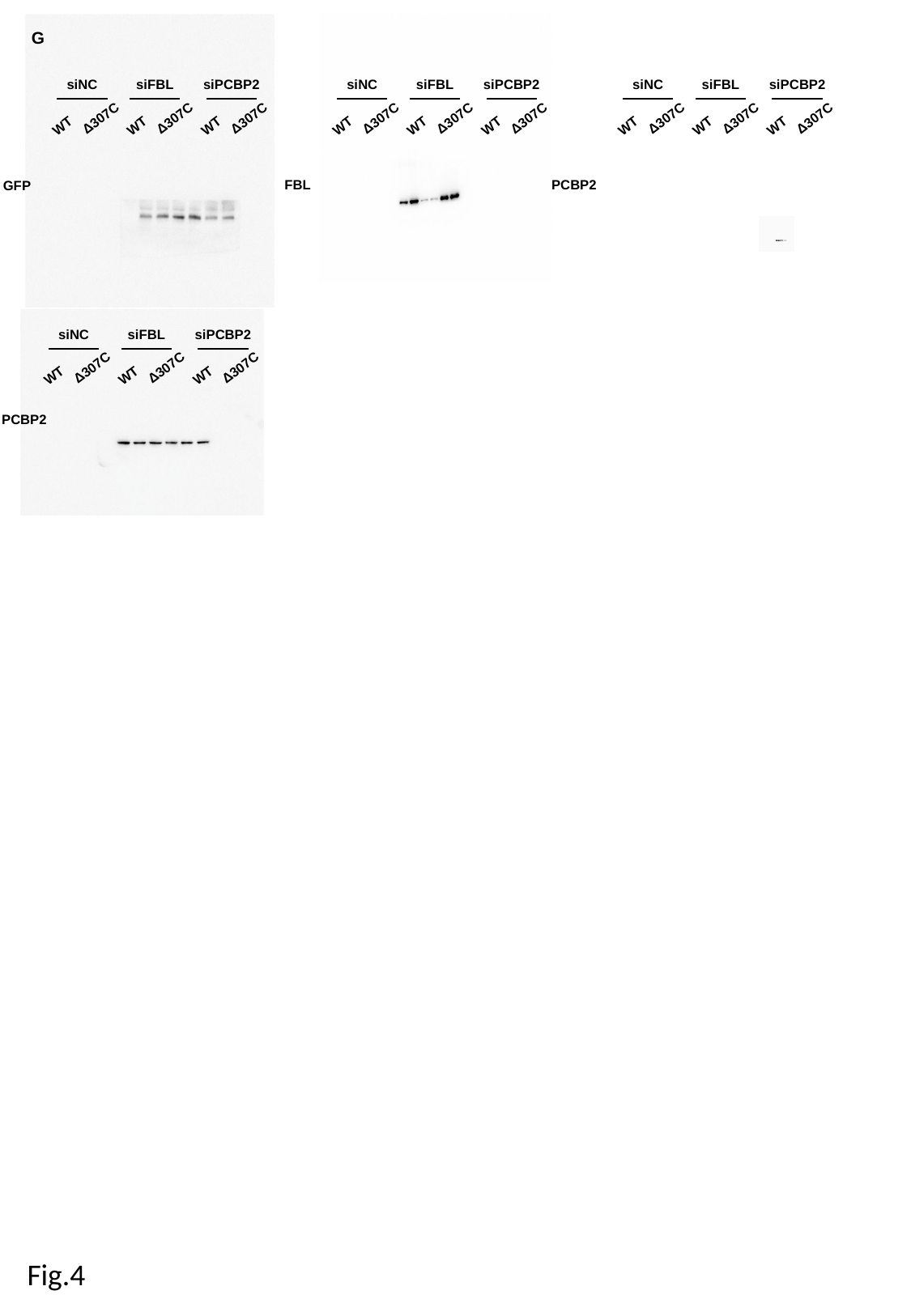

G
siNC
siFBL
siPCBP2
siNC
siFBL
siPCBP2
siNC
siFBL
siPCBP2
Δ307C
Δ307C
Δ307C
Δ307C
Δ307C
Δ307C
Δ307C
Δ307C
Δ307C
WT
WT
WT
WT
WT
WT
WT
WT
WT
FBL
PCBP2
GFP
siNC
siFBL
siPCBP2
Δ307C
Δ307C
Δ307C
WT
WT
WT
PCBP2
Fig.4

## Slide 6
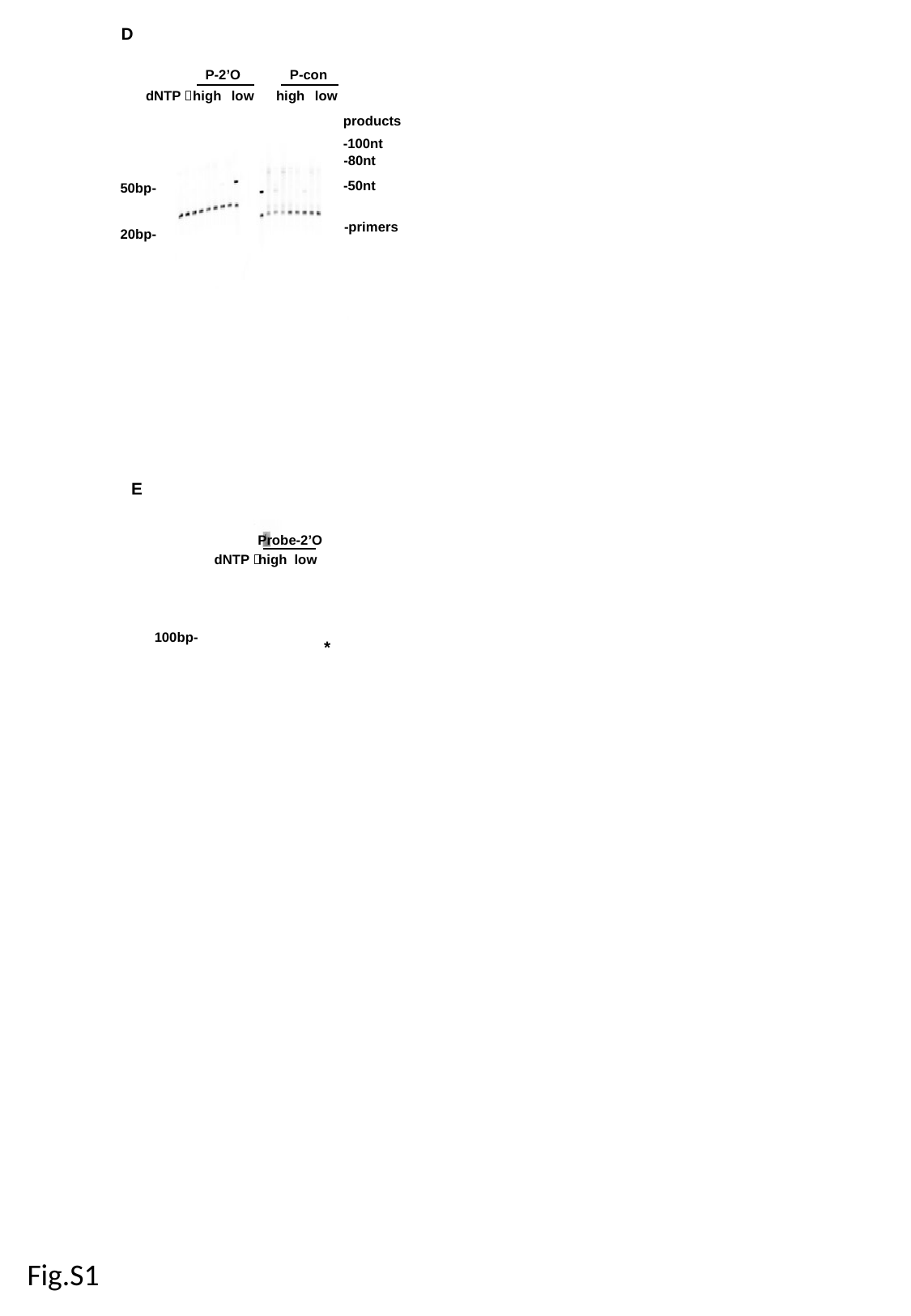

D
P-2’O
P-con
dNTP：
high
low
high
low
products
-100nt
-80nt
-50nt
50bp-
-primers
20bp-
E
Probe-2’O
dNTP：
high
low
100bp-
*
Fig.S1

## Slide 7
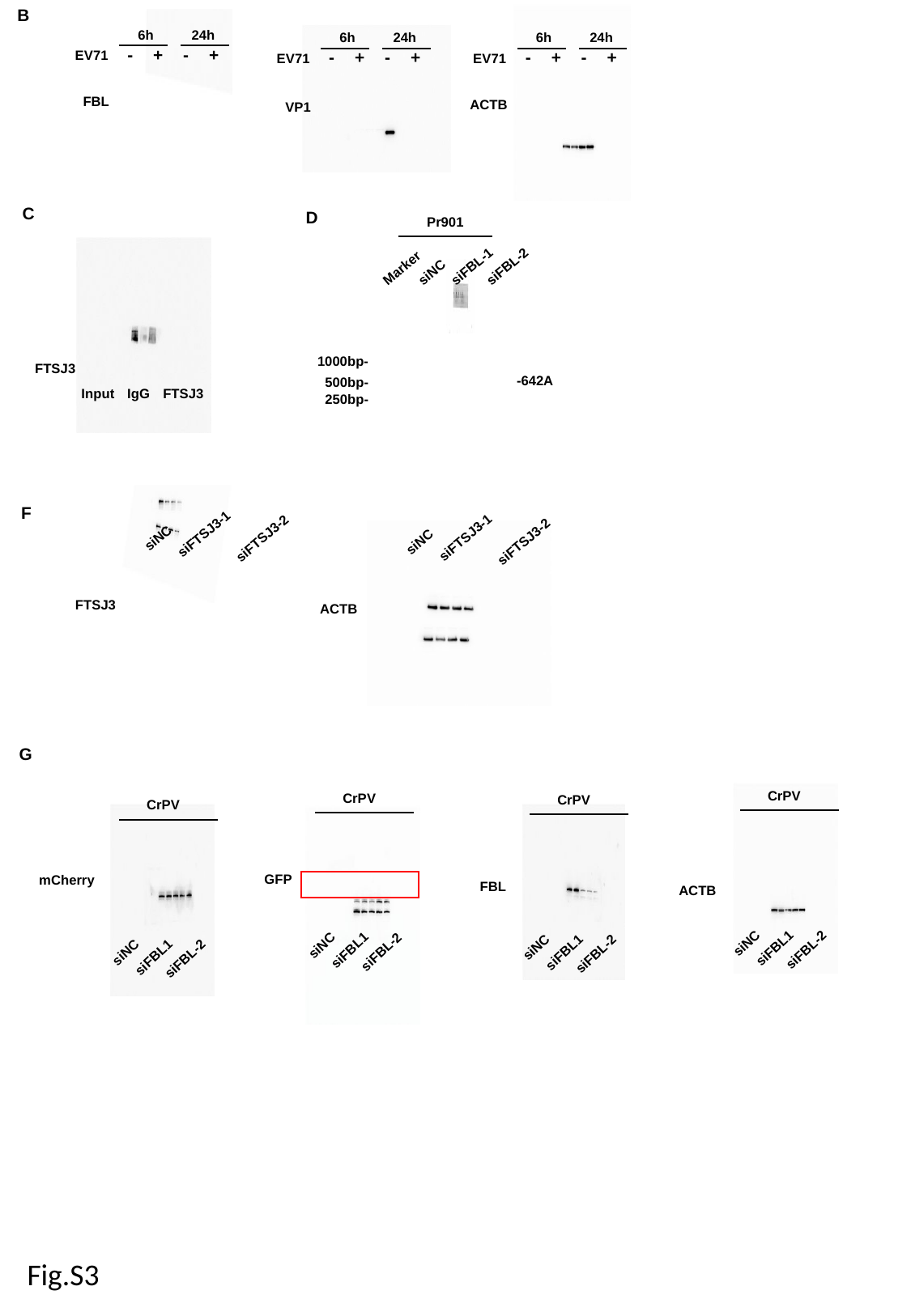

B
6h
24h
6h
24h
6h
24h
-+-+
-+-+
-+-+
EV71
EV71
EV71
FBL
ACTB
VP1
C
D
Pr901
siFBL-1
siFBL-2
Marker
siNC
1000bp-
FTSJ3
Input
IgG
FTSJ3
-642A
500bp-
250bp-
F
siFTSJ3-1
siNC
siFTSJ3-1
siFTSJ3-2
siNC
siFTSJ3-2
FTSJ3
ACTB
G
CrPV
CrPV
CrPV
CrPV
GFP
mCherry
FBL
ACTB
siNC
siFBL1
siFBL-2
siNC
siFBL1
siFBL-2
siNC
siFBL1
siFBL-2
siNC
siFBL1
siFBL-2
Fig.S3

## Slide 8
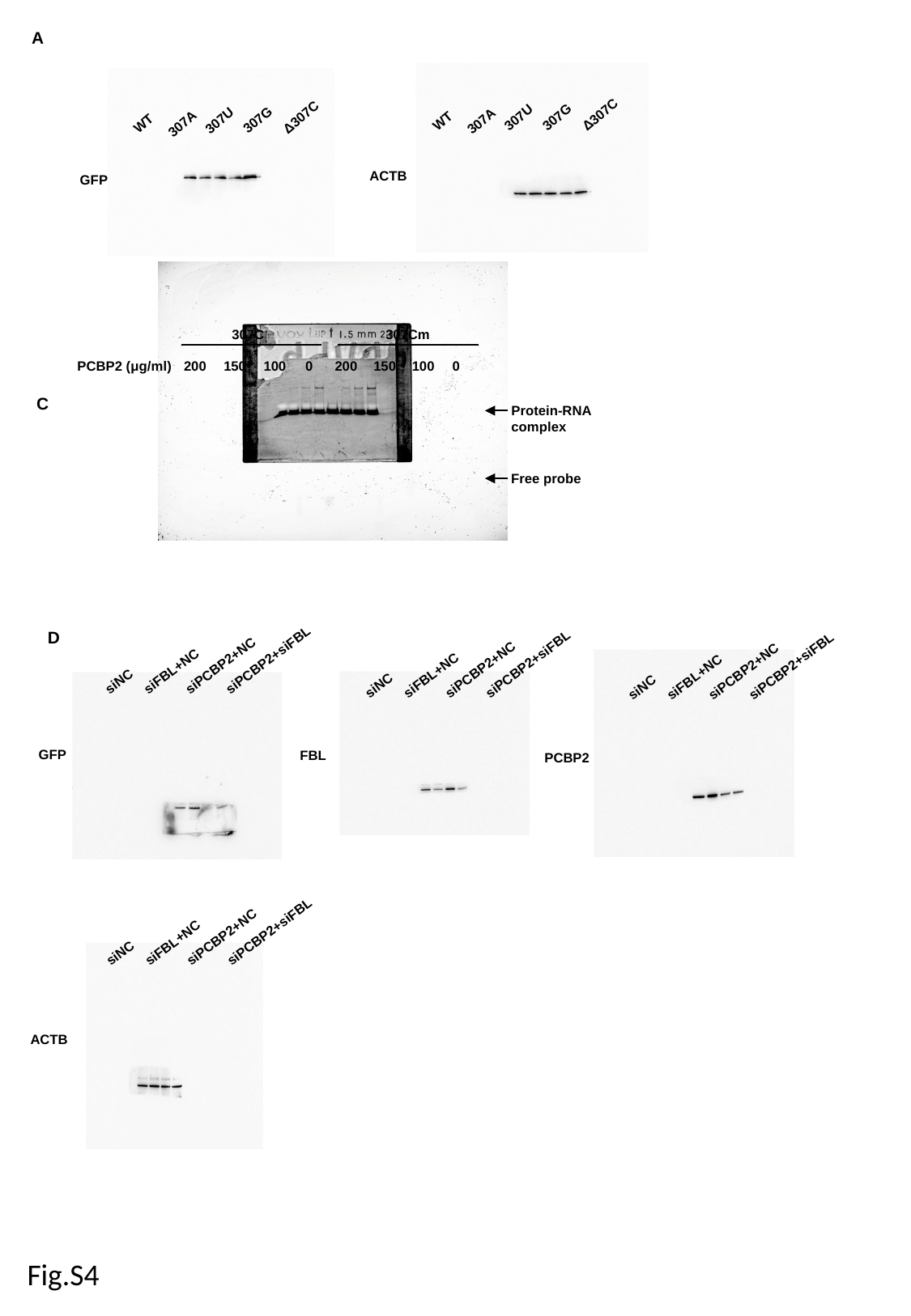

A
Δ307C
Δ307C
307G
307U
307G
307U
307A
WT
307A
WT
ACTB
GFP
307C
307Cm
PCBP2 (μg/ml)
200
150
100
0
200
150
100
0
C
Protein-RNA
complex
Free probe
D
siPCBP2+NC
siPCBP2+siFBL
siPCBP2+NC
siPCBP2+NC
siPCBP2+siFBL
siPCBP2+siFBL
siFBL+NC
siFBL+NC
siFBL+NC
siNC
siNC
siNC
GFP
FBL
PCBP2
siPCBP2+NC
siPCBP2+siFBL
siFBL+NC
siNC
ACTB
Fig.S4
