# Supplementary material for: Fibrillarin-mediated 2’-O-methylation serves as a translation brake on uncapped enterovirus RNA
Source: PLoS Pathog. 2026 Jul 20;22(7):e1014455. doi: 10.1371/journal.ppat.1014455 (PMC13405287; doi:10.1371/journal.ppat.1014455)
Supplement: Attachment — Submitted filename: Response-0612-2.docx [file ppat.1014455.s014.docx]

**PLOS Pathogens**

**Manuscript #: PPATHOGENS-D-26-00505**

**Title: Fibrillarin-mediated 2'-O-methylation serves as a translation brake on uncapped enterovirus RNA**

**Response to Reviewers**

**Part I - Summary**
Please use this section to discuss strengths/weaknesses of study, novelty/significance, general execution and scholarship.

Reviewer #1: Nm (2’-O-methylation) is one of the most abundant modifications in RNAs, including mRNA, rRNA, tRNA, snRNA, snoRNA, and miRNA. In eukaryotic cells, this modification was shown to be essential for accurate and efficient protein synthesis. However, the role of Nm in antivirus defense is poorly understood. The authors of this study investigated the role of host-produced Nm modifications in the translation, replication, and pathogenesis of enterovirus 71 (EV71). They developed a low-dNTP-based sequencing method (RACE-Nm), which, in combination with a machine learning-based predictor (NanoNm), allows for mapping Nm sites on RNAs at single-nucleotide resolution. Applying this method, the authors identified an important Nm site at 307C within the IRES. Methylation at 307C by the methyltransferase FBL prevented binding of PCBP2 to the IRES, and consequently inhibited viral translation. The reduced synthesis of the 3D polymerase was found to prevent activation of pro-viral host genes, resulting in impaired virus replication. Importantly, deletion of 307C enhanced virus replication and pathogenicity in mice.

Overall, this is an interesting study that demonstrates the important role of 2’-O-methylation at position 307C of the EV71 genome in suppressing virus translation and activating a novel host defense mechanism. The paper is technically sound, and the data support many conclusions. However, the study falls short in characterizing the effect of Nm at 307C on IRES activity, as some important controls are missing.

Response: Thank the reviewer for the positive assessment and constructive comments. Regarding several important controls and clarifications needed, several additional experiments and revisions were performed as suggested. Specifically, (1) added an acknowledgment that FBL may regulate EV71 translation through multiple Nm sites in Discussion; (2) included CrPV IRES as a negative control in the revised Fig. 3G and S3G; (3) introduced mCherry as a transfection efficiency control in the revised Fig. 3G and S3G; (4) performed EMSA with longer probes (50 nt) to confirm the inhibitory effect of 307Cm on PCBP2 binding in the revised Fig. S4C; (5) generated a double mutant (Δ307C/Δ642A) to test additive effects in the revised Fig. S6; (6) added commentary on the 3D-mediated host gene regulation in the Discussion. All figure legends and methods have been updated accordingly. The detailed revisions are shown below point by point.

Reviewer #2: This manuscript addresses the role of Fibrillarin-dependent RNA methylation for EV71 viral RNA replication. 2´-O-methylation (Nm) was analyzed using a sequencing method (RACE-Nm) in combination with a machine learning-based predictor (NanoNm). Authors report the methylation of several positions within the IRES element. Methylation of positions 307C and 642A inhibits viral RNA translation, reducing the levels of viral proteins. Authors concentrate on the effect of limited viral polymerase 3D in the activation of proviral host genes. They conclude that RNA methylation mediated by FBL, targeting IRES-dependent translation, represents a novel host defense mechanism. The manuscript is interesting and well written.

Response: Thank the reviewer for recognizing the interest of our work. In order to clarify the unexplored mechanism of 642A methylation and the potential contribution of other non-structural proteins, several experiments were performed and the manuscript was revised accordingly. Specifically, (1) acknowledged that the mechanism of 642A-mediated translational repression remains unknown and ruled out binding to PCBP1, PCBP2, EIF4G, and hnRNPK (Fig. 4E); (2) added a statement that other internal Nm sites within the coding region may also contribute to translational regulation in Discussion; (3) tested viral 3C protease and confirmed that it does not upregulate FOSL1, ITGB3, KCNQ4, while acknowledging that systematic testing of all non-structural proteins is needed. The manuscript has been revised accordingly. The detailed revisions are shown below point by point.

Reviewer #3: The manuscript by Zhang et al entitled, “Fibrillarin-mediated 2'-O-methylation serves as a translation brake on uncapped enterovirus RNA” describes an RNA modification in the IRES of EV71 that is added by a host enzyme, Fibrillarin. They present data to support their claim that this modification reduces the affinity for PCBP2, a protein that has been shown to be important for enterovirus translation. They present both binding data and a model for how this modification disrupts PCBP2 binding to the B loop. This is all very nice and straightforward work. Then they make the claim that “FBL suppresses EV71 replication through a 3Dpol-dependent host gene regulatory axis”. This claim is based on RNA-seq data from 2 different siRNAs against FBL, the first problem is that the overlap between the datasets is not good suggesting that one or both siRNAs have off target effects. Then, they looked at transcripts that were either up-regulated or down regulated at 24 hpi with EV71 (n=1) and from over 1000 transcript changes they found 12 overlapped with the FBL knockdown expression data. It seems to this reviewer that this data is not rigorous and maybe not even reproducible, but they continue to validate these 12 hits by performing siRNA KD and looking for the effects on viral RNA levels using RT-qPCR, which is surprising that they didn’t just measure viral titers, or viral protein by western. The RT-qPCR data has some problems discussed below given that the siNC is not clearly defined so most of what they may be observing could just be effects of an antiviral state due to the siRNA knockdowns and not specific to the genes that were targeted. Given the small effects, the lack of real biological replicates, and the failure to apply standards set out by MIQE, none of these changes in gene expression due to FBL knockdown or EV71 infection are likely to be significant or real. This means that the claim that these reductions in viral IRES activity – or 3Dpro viral protein production prevents activation of pro-viral host genes is not supported by the data. Nonetheless the strengths of this study are the improved method to identify Nm modifications, their data showing that a modification in the IRES functions to prevent PCBP2 binding reducing viral protein production and viral pathogenicity.

Response: Thank the reviewer for acknowledging the strengths of our study. To exclude the off-target effects, several additional experiments and revisions were performed as suggested. Specifically, (1) a third FBL-targeting siRNA (siFBL-3) was used to confirm that 8 of the 12 candidate genes were consistently regulated by all three siRNAs, with CCL5 being the only exception; (2) the siNC sequence and source have been added to Table S5; (3) viral titers (TCID₅₀) were measured under knockdown conditions of the four pro-viral genes in the revised Figure 5G; (4) key qPCR experiments have been repeated with three biological replicates in the revised Figure S5E. The detailed revisions are shown below point by point.

Major points:

The authors knockdown FBL and measure viral growth over 3 days. The legend should state what MOI the initial infections were performed at. Many of their assays do not state the MOI, but given that they seem to repeatedly rely on a 24 hpi time point for a lot of their assays, their MOI must be <1 because if they were using an MOI=1 then most of their cells should be more or less dead (PMID: 22039330 DOI: 10.3748/wjg.v17.i36.4135]), which means they are studying multiple rounds of infection. This needs to be clarified.

Response: The reviewer raises a valid point regarding the MOI. The experiments have been updated to indicate that infections were performed at an MOI of 0.1. A citation (reference 56) has been added to support the use of MOI = 0.1 to allow multiple rounds of infection. A representative statement has been added as follows (Lines 911-914):

“FBL knockdown was performed in RD cells using two independent siRNAs, and the cells were then infected with EV71 at an MOI of 0.1 to permit multiple rounds of infection (56).”

In Figure 4H, the authors show a model of the PCBP2 KH3 domain interacting with the B loop with either 307C or 307Cm and they claim that with the methylation there is a “reduction in the number of proximal amino acid residues was observed after modification (from 14 to 11 within 5 Å)”. However, this reviewer found it very difficult to see more than 1 interaction lost given the limited view of the image provided.

Response: Thank the reviewer for raising this concern regarding the visibility of the interaction changes. As suggested, we have provided a high-resolution picture of the structures (see separate .tif figure) to better illustrate the distinct binding modes. The residues within 5 Å of the RNA for both the unmodified and methylated structures were listed as follows. The residues within 5 Å of the unmodified 307C are: ASN13, LEU14, ILE15, CYS17, ILE18, ILE19, ARG21, GLN22, ALA24, LYS25, LYS12, VAL51, ILE53, and ASN68 (14 residues). Upon methylation, ILE15, VAL51, and ASN68 shift beyond 5 Å, reducing the count to 11 residues. Furthermore, the revised text now emphasizes conformational rearrangement rather than a simple loss of contacts (Lines 484-488):

"307Cm induces local conformational rearrangements in the RNA backbone and base orientation, leading to an altered interaction network with the PCBP2 KH3 domain, as evidenced by reduced proximal residues (from 14 to 11 within 5 Å) and a distinct binding interface."

Fig.4H. Comparative analysis of the binding interfaces between the KH3 domain and loop B. The interaction between the EV71 IRES loop and the PCBP2 KH3 domain was predicted using HelixFold3 (https://paddlehelix.baidu.com/app/all/helixfold3/forecast).

It is concerning that differential expression from two different siRNAs gave such a huge difference in altered gene expression with really a minority of genes overlapping. It seems like additional siRNAs should be tested as perhaps the siFBL-2 has some off target effects given that >3 times more genes were affected.

Response: Thank the reviewer for this important concern. To clarify this finding, an additional experiment was performed using a third FBL-targeting siRNA (siFBL-3) along with an additional negative siRNA control as suggested. Then, the expression of the 12 candidate genes were examined by qPCR under FBL knockdown conditions using all three siRNAs. The results showed that among the four initially identified pro-viral genes (FOSL1, ITGB3, KCNQ4, and TFPI2), three (FOSL1, ITGB3, and KCNQ4) were consistently regulated by all three siRNAs. Consequently, TFPI2 was removed from the list of pro-viral genes, and the manuscript has been revised to focus on the consistently regulated genes (FOSL1, ITGB3, and KCNQ4). The corresponding figures and text have been updated accordingly (Lines 501-505).

“Their regulation was confirmed by qPCR under both FBL-knockdown and infection conditions (Fig. S5D and S5E), and a third FBL-targeting siRNA (siFBL-3), along with an additional negative siRNA control, was tested to further rule out off-target effects. While the first two siRNAs consistently regulated all 12 candidate genes, the third siRNA showed inconsistent regulation for CCL5, TFPI2, and CREB3L1, indicating these are off-target hits.”

Fig. S5E. qPCR validation of the upregulated or downregulated host genes from panel Fig. 5B and 5C. A third FBL-targeting siRNA (siFBL-3), along with an additional negative siRNA control, was tested. GAPDH was used as internal normalization control.

For the siRNA knockdowns the authors do not define what their control (siNC) is. Is the control no siRNA or a control siRNA? If it is a non-targeting control RNA the authors must state what the sequence is or what the source of it is. This is important and speaks to whether the control is adequate to determine effects of an siRNA transfection as this alone can create an antiviral environment. This means that the decreases in viral production in figure 5G may be due to non-specific siRNA transfection and not because specific transcripts were targeted. It does appear that all the siRNA knockdowns result in very similar decreases in EV71 production suggesting this may be the case.

Response: Thank the reviewer for requesting clarification of the siRNA control. The siNC used in this study is a non-targeting control siRNA that has no significant sequence complementarity to any known human or viral gene. The sequence and source have now been added to Table S5. Specifically, siNC-1 sense: UUCUCCGAACGUGUCACGU(dT)(dT), antisense: ACGUGACACGUUCGGAGAA(dT)(dT); siNC-2 sense: GCGCGCUUUGUAGGAUUCG(dT)(dT), antisense: CGAAUCCUACAAAGCGCGC(dT)(dT).

Regarding the concern that siRNA transfection itself may induce nonspecific innate immune responses. If such nonspecific effects were the primary cause of the observed antiviral phenotypes, then the non-targeting control would be expected to create a similar antiviral environment as the gene-specific siRNAs, and all siRNAs should produce comparable levels of viral inhibition. Our data showed that siNC did not reduce viral RNA to the same extent as the gene-specific siRNAs (Fig. 5F). In the revised manuscript, we also measured viral titers, and siNC showed no reduction compared to the gene-specific siRNAs (revised Fig. 5G). Taken together, these data indicate that the observed antiviral effects are primarily due to specific gene knockdown rather than siRNA transfection-induced innate immunity.

Fig.5G. Viral titers (TCID₅₀) were measured under the knockdown conditions of the pro-viral genes. Knockdown of FOSL1, ITGB3, or KCNQ4 were performed in RD cells with two siRNAs for each gene, and the cells were infected with EV71 (MOI = 0.1). Viral titers in the supernatant were determined.

Figure S5D-E shows RT-qPCR to quantify changes in gene expression levels. However, most changes from the KD data are <2-fold and they are reported to be n=3 technical replicates not biological replicates. This data speaks to whether KD of the FBL mRNA really affects expression levels of these other transcripts and is used to support their claim that changes in gene expression from EV71 infection under conditions that limit viral gene expression (FBL present) has effects on expression of other host genes. GAPDH is used as a loading control, but probably they are using amplification of this mRNA as a control for RT-qPCR amplification.

Response: Thank the reviewer for raising this concern regarding biological replicates. As suggested, three biological samples for each knockdown condition have been now prepared when adding the new siFBL-3. The revised data represent the average of three samples in each knockdown condition. Since its regulation of specific host mRNAs is likely indirect, through downstream effectors such as 3D-mediated pathways, this may explain the subtle expression changes.

GAPDH was used as an internal normalization control to correct for variations in RNA input in RT-qPCR. Our original description in the legend of Figure S5D-E has been revised accordingly.

**Part II – Major Issues: Key Experiments Required for Acceptance**
Please use this section to detail the key new experiments or modifications of existing experiments that should be absolutely required to validate study conclusions.

Generally, there should be no more than 3 such required experiments or major modifications for a "Major Revision" recommendation. If more than 3 experiments are necessary to validate the study conclusions, then you are encouraged to recommend "Reject".

Reviewer #1: 1. Lines 398-403 and Figs. 3F, G. Global inhibition of Nm by FBL knockdown may enhance EV71 translation and EV71 IRES-directed GFP synthesis not only by reducing methylation at 307C in the IRES. As they showed, the absence of methylation at 642A also increases translation (Fig. 4B). Moreover, it is known that the translation of 2’-methylated codons is severely impaired, and FBL knockdown would be expected to relieve this inhibition (ref. 6). So, the effects of FBL on EV71 translation cannot be interpreted unequivocally. This should be acknowledged.

Response: Thank the reviewer for this insightful comment. Accordingly, an acknowledgment has been added in the Discussion section (Lines 602-613) as follows:

" Nevertheless, FBL knockdown enhances EV71 translation through multiple mechanisms. Beyond the C307 site, loss of methylation at A642 also promotes IRES activity (Fig. 4B). However, the mechanism underlying this translational repression remains unknown. Pulldown assays ruled out binding to PCBP1, PCBP2, EIF4G, and hnRNPK (Fig. 4E), suggesting that A642 acts through a distinct mechanism. In addition to the 5'UTR, NanoNm analysis identified numerous Nm sites within the EV71 coding region. Given that Nm within coding regions can impair translation elongation in cellular mRNAs (6), it is plausible that similar mechanisms operate on the viral genome. Future studies are needed to explore this possibility. Therefore, the translational enhancement upon FBL depletion may reflect the cumulative loss of Nm marks across the viral genome, not solely the 307C site."

2. Fig. 3G. A negative control is required. Would FBL knockdown affect the translation from IRESs that do not use PCBP2 (e.g., CrPV IRES)?

Response: Thank the reviewer for this suggestion. In order to address this question, we examined the effect of FBL knockdown on the CrPV (Cricket paralysis virus) IRES as suggested. The CrPV IRES (AF218039.1, IRES 302-708) was inserted between the CMV promoter and the GFP gene, using the same backbone as the EV71 IRES reporter. As shown in revised Figure 3G and new S3G, FBL knockdown had no significant effect on CrPV IRES-driven GFP expression, whereas it enhanced EV71 IRES activity under the same conditions. These results confirm that the regulatory effect of FBL is not a general effect on IRES-dependent translation and is likely mediated through specific internal Nm modifications on the EV71 IRES. The corresponding figure legend has been updated accordingly.

Fig. 3G. FBL-mediated suppression of IRES activity inhibits 3D protein synthesis. Western blot analysis indicated significantly enhanced GFP expression from the EV71 IRES reporter following FBL knockdown in 293T cells. GFP/actin ratios are shown below the image. An mCherry reporter was co-transfected as a transfection efficiency control.

Fig. S3G. The CrPV IRES (Cricket paralysis virus, AF218039.1, IRES 302-708) was inserted between the CMV promoter and the GFP gene, with the same backbone as the EV71 IRES reporter. FBL knockdown did not significantly alter GFP expression from this reporter. An mCherry reporter was co-transfected as a transfection control.

3. Figs. 3G, 4B, 4G, S4A, and S4C require controls for transfection efficiency.

Response: Thank the reviewer for this concern. To control for transfection efficiency, an independent mCherry reporter without IRES was co-transfected as an internal control in revised Fig. 3G and Fig. S3G. The mCherry signal was comparable between control and FBL knockdown groups, confirming that transfection efficiency was consistent across conditions. The revised data and figure legends have been updated accordingly in the manuscript.

For Figures 4B, 4G, S4A, and S4C, these experiments were performed using the same optimized transfection protocol and conditions as in Fig. 3G. Given that all transfections were carried out in the same cell line with comparable DNA amounts and reagents, and that the mCherry control in Fig. 3G demonstrated consistent efficiency, the transfection efficiency was similarly reliable across these experiments.

4. Fig.4E. The probes bearing 307C that are used for the pull-down assay are only 25-nucleotide long. The binding of proteins to a 25mer can be different than binding to the 25mer within a longer sequence context. The authors should confirm that the PCBP2 protein binds differently to longer probes (e.g., >50nt) that contain or do not contain the 307C modification. Could they also perform binding affinity measurements and determine Kd values for PCBP2 and these probes by MicroScale Thermophoresis (MST) or Gel-shift assay (EMSA)?

Response: Thank the reviewer for this excellent suggestion. As suggested, EMSA assays were performed using longer RNA probes (50 nt) containing either unmodified 307C or 2'-O-methylated 307Cm (sequences listed in revised Table S5). Flag-tagged PCBP2 was overexpressed in 293T cells, affinity-purified using anti-Flag beads, and eluted with 3× Flag peptide. The eluted protein was quantified and used in binding reactions at four gradient concentrations. As shown in revised Fig. S4C, the 307Cm modification consistently reduced PCBP2 complex formation compared to the unmodified 307C probe across all protein concentrations tested. These data are not only consistent with previous reports that PCBP2 binding depends on the consecutive 6C-tract in loop B, but also confirm that the inhibitory effect observed with the short 25-mer probes is not an artifact of probe length. Furthermore, these results support our conclusion that 307Cm impairs PCBP2 recruitment to the EV71 IRES. Because our EMSA was performed using affinity purified Flag-PCBP2 eluted from anti-Flag beads, the protein preparation contained a mixture of active and inactive PCBP2 as well as trace contaminants. Without further purification to homogeneity and accurate determination of active protein concentration, calculation of an absolute Kd would be unreliable.

Fig. S4C. EMSA analysis of PCBP2 binding to FAM-labeled RNA probes containing either unmodified 307C or 2'-O-methylated 307Cm. Flag-tagged PCBP2 was overexpressed in 293T cells and eluted from anti-Flag beads using 3× Flag peptide. The eluted protein was quantified and used at three gradient concentrations in the binding reactions. The 307Cm modification consistently reduced PCBP2 complex formation compared to the unmodified 307C probe across all concentrations tested. Free probes and PCBP2-RNA complexes are indicated. The right panel shows the quantification of bound RNA calculated as the percentage of shifted RNA to free probes.

5. Fig. 6. Deletion of 307Cm or 642Cm enhances EV71 replication and pathogenicity in mice. Would the deletion of both 307Cm and 642Cm amplify the effects?

Response: Thank the reviewer for this insightful question. To investigate whether deletion of both Nm sites produces additive effects, we generated a double mutant (Δ307C/Δ642A) and compared its pathogenicity with the Δ307C single mutant in AG6 mice. As shown in revised Figure S6, the double mutant exhibited modestly accelerated mortality kinetics compared to the Δ307C single mutant but did not significantly alter viral RNA loads in various organs. These results suggest that the two Nm sites may function independently, possibly because both act through the same IRES-dependent translation pathway or due to ceiling effects in the mouse model.

Figure S6. Comparison of pathogenicity between Δ307C and Δ307C/Δ642A double mutant. (A) Survival curves of AG6 mice infected with EV71 Δ307C or Δ307C/Δ642A double mutant virus (n = 7 per group). (B) Average body weight change of infected mice over time. (C) Viral RNA loads in muscle, intestine, and brain at 3 days post-infection, as measured by qRT-PCR (n = 6 per group).

6. The mechanism behind upregulation of pro-virus gene expression by 3D is unclear. It is also uncertain whether the enhancement of 3D synthesis resulting from the deletion of 307C is sufficient to activate synthesis of the pro-virus proteins. Finally, what might be the mechanism of EV71 replication enhancement by these proteins? Although addressing these questions experimentally is beyond the scope of this article, some commentaries are required.

Response: Thank the reviewer for raising these mechanistic questions. As suggested, a commentary paragraph has been added in the Discussion section (Lines 649-655) following the sentence "Despite these advances, several intriguing questions remain":

“A key question is how 3D upregulates FOSL1, ITGB3, and KCNQ4, and what their functional roles are in the viral life cycle. Given the around 10-fold induction of FOSL1 upon 3D expression, transcriptional activation is proposed. Subsequently, FOSL1 may promote other host factors for viral replication, while ITGB3 could facilitate viral entry or signaling. Although our data suggest that increased 3D synthesis from the Δ307C mutant may drive this upregulation, direct causality requires future study.”

Reviewer #2: Out of 251 high-confidence Nm sites, only 3 positions (307, 528, and 758) are recognized as bona fide methylated residues by NanoNm method. However, 642A remains as a functionally relevant methylated position, despite it was not covered by the second approach. Author´s then show that FBL-dependent methylation at positions 307 and 642 within the IRES element inhibit viral RNA translation (Figs 3 and 4). The effect of 307 methylation was analyzed in detail, but surprisingly, the mechanistic basis of inhibition mediated by methylated 642A is not further studied, and there are not comments about this.
Response: Thank the reviewer for this important comment. The mechanistic basis for the 642A Nm site remains unexplored. Unlike the 307C site, which lies within a well-characterized PCBP2-binding poly-C tract in stem-loop IV, the 642A region has no previously reported binding proteins to our knowledge. Identifying the binding partners for this region is essential to understanding the mechanism. RNA pulldown assays were performed using a short RNA oligo containing 2'-O-methylation at 642A to examine the binding potential of known IRES-binding proteins, including PCBP1, PCBP2, EIF4G, and hnRNPK. No significant difference in binding was observed for any of these factors (revised Fig. 4E). Therefore, identifying the binding partners will require unbiased approaches such as mass spectrometry based RNA pull-down and crosslinking immunoprecipitation, followed by functional validation. This important work remains for future investigation. To address the reviewer's concern, we have added the following to the Discussion (Lines 602-607):

" Nevertheless, FBL knockdown enhances EV71 translation through multiple mechanisms. Beyond the C307 site, loss of methylation at A642 also promotes IRES activity (Fig. 4B). However, the mechanism underlying this translational repression remains unknown. Pulldown assays ruled out binding to PCBP1, PCBP2, EIF4G, and hnRNPK (Fig. 4E), suggesting that A642 acts through a distinct mechanism. "

Fig. 4E. RNA pulldown assay demonstrates that 307Cm impairs PCBP2 binding. Biotin-labeled RNA oligonucleotides, either unmodified or containing a Cm at position 307, were used for the assays, with oligonucleotides containing 642A or 642Am serving as controls. Bound PCBP2 was detected by western blot. Binding of PCBP1, EIF4G, and hnRNPK were also assessed.

Related to the implications of overall viral genome methylation in infected cells, most marks are observed within the coding region of the EV71 RNA (Fig 1B). These viral RNA modifications may alter virus replication directly. This manuscript shows that FBL overexpression promotes methylation of the IRES region (Figs 3 and 4). However, it remains possible that other positions on the viral RNA could be methylated by FBL. I could not find if this possibility was addressed.
Response: Thank the reviewer for these thoughtful comments. While the current study focused on functionally characterizing Nm sites within the 5'UTR due to their regulatory potential, we fully acknowledge that other internal Nm sites, including those within the coding region, may also influence viral replication, the following sentence has been added to the Discussion (Lines608-614) to clarify this point:

" In addition to the 5'UTR, NanoNm analysis identified numerous Nm sites within the EV71 coding region. Given that Nm within coding regions can impair translation elongation in cellular mRNAs (6), it is plausible that similar mechanisms operate on the viral genome. Future studies are needed to explore this possibility. Therefore, the translational enhancement upon FBL depletion may reflect the cumulative loss of Nm marks across the viral genome, not solely the 307C site."

Authors focused on viral 3D protein expression as a way to analyze the role of FBL-dependent methylation of host genes. 3D expression in uninfected cells, using VP1 in parallel, revealed transcriptional upregulation of four genes (FOSL1, ITGB3, KCNQ4, and TFPI2). Authors claim that expression of these genes is associated with limited 3D protein synthesis upon FBL-mediated methylation of the IRES element (Fig 5). This conclusion is not directly supported by the results. The limited expression of other non-structural proteins, with potential effect on antiviral response, could also be responsible for this effect.

Response: Thank the reviewer for this important comment. To begin addressing this possibility, the viral 3C protease was tested and found that its expression did not upregulate these four genes as shown below, suggesting that the observed transcriptional activation is not a general consequence of all non-structural proteins.

Figure for Reviewers. qPCR analysis of host gene expression in 293T cells overexpressing EV71 3C, showing regulation of candidate genes identified in Figure 5C.

In addition, we acknowledge that systematic testing of all non-structural proteins (2A, 2B, 2C, 3A, 3B) would be required to definitively rule out contributions from other viral factors. A statement of this limitation has been added to the Discussion (Lines 655-658):

"We cannot formally exclude the possibility that other viral non-structural proteins may also contribute to the upregulation of FOSL1, ITGB3, and KCNQ4. Systematic testing of all non-structural proteins would be required to establish the specificity of 3D in this process."

Reviewer #3: (No Response)

**Part III – Minor Issues: Editorial and Data Presentation Modifications**
Please use this section for editorial suggestions as well as relatively minor modifications of existing data that would enhance clarity.

Reviewer #1: 1. Figs. 3G and 4B, G. Could they quantify GFP expression (preferably by measuring fluorescence), as some effects are very small (e.g., Fig. 4G)?

Response: Thank the reviewer for this suggestion. GFP fluorescence intensities have been quantified now. The relative data normalized to the control have been added below each image in revised Figures 3G, 4B, and 4G. This has been described in the figure legends accordingly.

Fig. 3G. FBL-mediated suppression of IRES activity inhibits 3D protein synthesis. Western blot analysis indicated significantly enhanced GFP expression from the EV71 IRES reporter following FBL knockdown in 293T cells. GFP/actin ratios are shown below the image. An mCherry reporter was co-transfected as a transfection efficiency control.

Fig. 4B. Effect of Nm site deletion mutations on IRES-mediated translation. GFP expression from the EV71 IRES reporter construct (as in Fig. 3G) was measured in cells transfected with wild-type or mutant reporters. GFP/actin ratios are shown below the image.

Fig. 4G. GFP expression from the WT or mutant EV71 IRES reporters was measured in 293T cells under the indicated knockdown conditions (siPCBP2 and/or siFBL). GFP/actin ratios are shown below the image.

2. In Fig. 4D (but not in Fig. 3F) VP1 migrates as a double band. Why?

Response: Thank the reviewer. A smaller band migrating below VP1 could be seen in other experiments, likely due to partial proteolytic cleavage during sample preparation. For example, a faint band is also visible in Figure 3F (right panel, overexpression condition). In Figure 4D, this band appears stronger, which may be due to differences in the specific experimental conditions.

Reviewer #2: -

Reviewer #3: Typos:
Line 84 delete ‘our’
line 431 – should be poly not ploy
line 448 – capitalization
Formatting 117-121

Respones: Thank the reviewer for carefully reading the manuscript. All of these typos have been corrected, and other typos identified during revision have also been corrected throughout the manuscript.
