# Supplementary material for: Fibrillarin-mediated 2’-O-methylation serves as a translation brake on uncapped enterovirus RNA
Source: PLoS Pathog. 2026 Jul 20;22(7):e1014455. doi: 10.1371/journal.ppat.1014455 (PMC13405287; doi:10.1371/journal.ppat.1014455)
Supplement: Attachment — Submitted filename: Response-0703.docx [file ppat.1014455.s015.docx]

**PLOS Pathogens**

**Manuscript #: PPATHOGENS-D-26-00505R1**

**Title: Fibrillarin-mediated 2'-O-methylation serves as a translation brake on uncapped enterovirus RNA**

**Response to Reviewers**

Reviewer #1: The authors suggest that the FBL-mediated attenuation of viral infection is due to the prevention of 3D-dependent upregulation of pro-viral host genes. However, the reduced efficiency of viral infection might also result from the general inhibition of viral protein synthesis. This should be mentioned in the Discussion.

Response: Thank the reviewer for this suggestion. We have added the following statement to the Discussion section (lines 649-652):

“First, it is worth noting that FBL-mediated attenuation of viral infection may result not only from inhibition of 3D-dependent upregulation of pro-viral host genes but also from suppression of viral protein synthesis.”
