# Supplementary material for: Fibrillarin-mediated 2’-O-methylation serves as a translation brake on uncapped enterovirus RNA
Source: PLoS Pathog. 2026 Jul 20;22(7):e1014455. doi: 10.1371/journal.ppat.1014455 (PMC13405287; doi:10.1371/journal.ppat.1014455)
Supplement: Peer review history

# Peer review: Decision Letter 0

**DOI:** 10.1371/journal.ppat.1014455.r001

**Authors:** Alexander Gorbalenya, Peter Sarnow

27 Mar 2026

-->PPATHOGENS-D-26-00505

Fibrillarin-mediated 2'-O-methylation serves as a translation brake on uncapped enterovirus RNA

PLOS Pathogens

Dear Dr. Liu,

Thank you for submitting your manuscript to PLOS Pathogens. After careful consideration, we feel that it has merit but does not fully meet PLOS Pathogens's publication criteria as it currently stands. Therefore, we invite you to submit a revised version of the manuscript that addresses the points raised during the review process.

While all three reviewers noted the importance of your study, they all raised numerous concerns that need to be carefully addressed. I agree with reviewer #3 that effects on viral titers need to be examined.

Please submit your revised manuscript by May 26 2026 11:59PM. If you will need more time than this to complete your revisions, please reply to this message or contact the journal office at plospathogens@plos.org. When you're ready to submit your revision, log on to https://www.editorialmanager.com/ppathogens/ and select the 'Submissions Needing Revision' folder to locate your manuscript file.

Please include the following items when submitting your revised manuscript:

* A letter that responds to each point raised by the editor and reviewer(s). You should upload this letter as a separate file labeled 'Response to Reviewers'. This file does not need to include responses to any formatting updates and technical items listed in the 'Journal Requirements' section below.

* A marked-up copy of your manuscript that highlights changes made to the original version. You should upload this as a separate file labeled 'Revised Manuscript with Track Changes'.

* An unmarked version of your revised paper without tracked changes. You should upload this as a separate file labeled 'Manuscript'.

If you would like to make changes to your financial disclosure, competing interests statement, or data availability statement, please make these updates within the submission form at the time of resubmission. Guidelines for resubmitting your figure files are available below the reviewer comments at the end of this letter.

We look forward to receiving your revised manuscript.

Kind regards,

Peter Sarnow

Academic Editor

PLOS Pathogens

Alexander Gorbalenya

Section Editor

PLOS Pathogens

Sumita Bhaduri-McIntosh

Editor-in-Chief

PLOS Pathogens

orcid.org/0000-0003-2946-9497

Michael Malim

Editor-in-Chief

PLOS Pathogens

orcid.org/0000-0002-7699-2064

**Journal Requirements:**

1) Please ensure that the CRediT author contributions listed for every co-author are completed accurately and in full.

At this stage, the following Authors/Authors require contributions: Kui Zhang, Xuliu Zhou, Baocheng Yu, Xiang Guo, Li Zuo, Lishi Liu, Zhen Chen, Xueyan Zhang, Haojie Hao, Huanzhou Xu, Fang Huang, Wuxiang Guan, and Haibin Liu. Please ensure that the full contributions of each author are acknowledged in the "Add/Edit/Remove Authors" section of our submission form.

The list of CRediT author contributions may be found here: https://journals.plos.org/plospathogens/s/authorship#loc-author-contributions

2) Please provide an Author Summary. This should appear in your manuscript between the Abstract (if applicable) and the Introduction, and should be 150-200 words long. The aim should be to make your findings accessible to a wide audience that includes both scientists and non-scientists. Sample summaries can be found on our website under Submission Guidelines:

https://journals.plos.org/plospathogens/s/submission-guidelines#loc-parts-of-a-submission

3) We do not publish any copyright or trademark symbols that usually accompany proprietary names, eg ©,  ®, or TM  (e.g. next to drug or reagent names). Therefore please remove all instances of trademark/copyright symbols throughout the text, including:

- ® on page: 6 and 10.

4) Please upload all main figures as separate Figure files in .tif or .eps format. For more information about how to convert and format your figure files please see our guidelines:

https://journals.plos.org/plospathogens/s/figures

5) We have noticed that you have uploaded Supporting Information files, but you have not included a list of legends. Please add a full list of legends for your Supporting Information files after the references list.

**Reviewers' Comments:**

Reviewer's Responses to Questions

**Part I - Summary**

Please use this section to discuss strengths/weaknesses of study, novelty/significance, general execution and scholarship.

Reviewer #1: Nm (2’-O-methylation) is one of the most abundant modifications in RNAs, including mRNA, rRNA, tRNA, snRNA, snoRNA, and miRNA. In eukaryotic cells, this modification was shown to be essential for accurate and efficient protein synthesis. However, the role of Nm in antivirus defense is poorly understood. The authors of this study investigated the role of host-produced Nm modifications in the translation, replication, and pathogenesis of enterovirus 71 (EV71). They developed a low-dNTP-based sequencing method (RACE-Nm), which, in combination with a machine learning-based predictor (NanoNm), allows for mapping Nm sites on RNAs at single-nucleotide resolution. Applying this method, the authors identified an important Nm site at 307C within the IRES. Methylation at 307C by the methyltransferase FBL prevented binding of PCBP2 to the IRES, and consequently inhibited viral translation. The reduced synthesis of the 3D polymerase was found to prevent activation of pro-viral host genes, resulting in impaired virus replication. Importantly, deletion of 307C enhanced virus replication and pathogenicity in mice.

Overall, this is an interesting study that demonstrates the important role of 2’-O-methylation at position 307C of the EV71 genome in suppressing virus translation and activating a novel host defense mechanism. The paper is technically sound, and the data support many conclusions. However, the study falls short in characterizing the effect of Nm at 307C on IRES activity, as some important controls are missing.

Reviewer #2: This manuscript addresses the role of Fibrillarin-dependent RNA methylation for EV71 viral RNA replication. 2´-O-methylation (Nm) was analyzed using a sequencing method (RACE-Nm) in combination with a machine learning-based predictor (NanoNm). Authors report the methylation of several positions within the IRES element. Methylation of positions 307C and 642A inhibits viral RNA translation, reducing the levels of viral proteins. Authors concentrate on the effect of limited viral polymerase 3D in the activation of proviral host genes. They conclude that RNA methylation mediated by FBL, targeting IRES-dependent translation, represents a novel host defense mechanism. The manuscript is interesting and well written.

Reviewer #3: The manuscript by Zhang et al entitled, “Fibrillarin-mediated 2'-O-methylation serves as a translation brake on uncapped enterovirus RNA” describes an RNA modification in the IRES of EV71 that is added by a host enzyme, Fibrillarin. They present data to support their claim that this modification reduces the affinity for PCBP2, a protein that has been shown to be important for enterovirus translation. They present both binding data and a model for how this modification disrupts PCBP2 binding to the B loop. This is all very nice and straightforward work. Then they make the claim that “FBL suppresses EV71 replication through a 3Dpol-dependent host gene regulatory axis”. This claim is based on RNA-seq data from 2 different siRNAs against FBL, the first problem is that the overlap between the datasets is not good suggesting that one or both siRNAs have off target effects. Then, they looked at transcripts that were either up-regulated or down regulated at 24 hpi with EV71 (n=1) and from over 1000 transcript changes they found 12 overlapped with the FBL knockdown expression data. It seems to this reviewer that this data is not rigorous and maybe not even reproducible, but they continue to validate these 12 hits by performing siRNA KD and looking for the effects on viral RNA levels using RT-qPCR, which is surprising that they didn’t just measure viral titers, or viral protein by western. The RT-qPCR data has some problems discussed below given that the siNC is not clearly defined so most of what they may be observing could just be effects of an antiviral state due to the siRNA knockdowns and not specific to the genes that were targeted. Given the small effects, the lack of real biological replicates, and the failure to apply standards set out by MIQE, none of these changes in gene expression due to FBL knockdown or EV71 infection are likely to be significant or real. This means that the claim that these reductions in viral IRES activity – or 3Dpro viral protein production prevents activation of pro-viral host genes is not supported by the data. Nonetheless the strengths of this study are the improved method to identify Nm modifications, their data showing that a modification in the IRES functions to prevent PCBP2 binding reducing viral protein production and viral pathogenicity.

Major points:

The authors knockdown FBL and measure viral growth over 3 days. The legend should state what MOI the initial infections were performed at. Many of their assays do not state the MOI, but given that they seem to repeatedly rely on a 24 hpi time point for a lot of their assays, their MOI must be <1 because if they were using an MOI=1 then most of their cells should be more or less dead (PMID: 22039330 DOI: 10.3748/wjg.v17.i36.4135]), which means they are studying multiple rounds of infection. This needs to be clarified.

In Figure 4H, the authors show a model of the PCBP2 KH3 domain interacting with the B loop with either 307C or 307Cm and they claim that with the methylation there is a “reduction in the number of proximal amino acid residues was observed after modification (from 14 to 11 within 5 Å)”. However, this reviewer found it very difficult to see more than 1 interaction lost given the limited view of the image provided.

It is concerning that differential expression from two different siRNAs gave such a huge difference in altered gene expression with really a minority of genes overlapping. It seems like additional siRNAs should be tested as perhaps the siFBL-2 has some off target effects given that >3 times more genes were affected.

For the siRNA knockdowns the authors do not define what their control (siNC) is. Is the control no siRNA or a control siRNA? If it is a non-targeting control RNA the authors must state what the sequence is or what the source of it is. This is important and speaks to whether the control is adequate to determine effects of an siRNA transfection as this alone can create an antiviral environment. This means that the decreases in viral production in figure 5G may be due to non-specific siRNA transfection and not because specific transcripts were targeted. It does appear that all the siRNA knockdowns result in very similar decreases in EV71 production suggesting this may be the case.

Figure S5D-E shows RT-qPCR to quantify changes in gene expression levels. However, most changes from the KD data are <2-fold and they are reported to be n=3 technical replicates not biological replicates. This data speaks to whether KD of the FBL mRNA really affects expression levels of these other transcripts and is used to support their claim that changes in gene expression from EV71 infection under conditions that limit viral gene expression (FBL present) has effects on expression of other host genes. GAPDH is used as a loading control, but probably they are using amplification of this mRNA as a control for RT-qPCR amplification.

**********

**Part II – Major Issues: Key Experiments Required for Acceptance**

Please use this section to detail the key new experiments or modifications of existing experiments that should be absolutely required to validate study conclusions.

Generally, there should be no more than 3 such required experiments or major modifications for a "Major Revision" recommendation. If more than 3 experiments are necessary to validate the study conclusions, then you are encouraged to recommend "Reject".

Reviewer #1: 1. Lines 398-403 and Figs. 3F, G. Global inhibition of Nm by FBL knockdown may enhance EV71 translation and EV71 IRES-directed GFP synthesis not only by reducing methylation at 307C in the IRES. As they showed, the absence of methylation at 642A also increases translation (Fig. 4B). Moreover, it is known that the translation of 2’-methylated codons is severely impaired, and FBL knockdown would be expected to relieve this inhibition (ref. 6). So, the effects of FBL on EV71 translation cannot be interpreted unequivocally. This should be acknowledged.

2. Fig. 3G. A negative control is required. Would FBL knockdown affect the translation from IRESs that do not use PCBP2 (e.g., CrPV IRES)?

3. Figs. 3G, 4B, 4G, S4A, and S4C require controls for transfection efficiency.

4. Fig.4E. The probes bearing 307C that are used for the pull-down assay are only 25-nucleotide long. The binding of proteins to a 25mer can be different than binding to the 25mer within a longer sequence context. The authors should confirm that the PCBP2 protein binds differently to longer probes (e.g., >50nt) that contain or do not contain the 307C modification. Could they also perform binding affinity measurements and determine Kd values for PCBP2 and these probes by MicroScale Thermophoresis (MST) or Gel-shift assay (EMSA)?

5. Fig. 6. Deletion of 307Cm or 642Cm enhances EV71 replication and pathogenicity in mice. Would the deletion of both 307Cm and 642Cm amplify the effects?

6. The mechanism behind upregulation of pro-virus gene expression by 3D is unclear. It is also uncertain whether the enhancement of 3D synthesis resulting from the deletion of 307C is sufficient to activate synthesis of the pro-virus proteins. Finally, what might be the mechanism of EV71 replication enhancement by these proteins? Although addressing these questions experimentally is beyond the scope of this article, some commentaries are required.

Reviewer #2: Out of 251 high-confidence Nm sites, only 3 positions (307, 528, and 758) are recognized as bona fide methylated residues by NanoNm method. However, 642A remains as a functionally relevant methylated position, despite it was not covered by the second approach. Author´s then show that FBL-dependent methylation at positions 307 and 642 within the IRES element inhibit viral RNA translation (Figs 3 and 4). The effect of 307 methylation was analyzed in detail, but surprisingly, the mechanistic basis of inhibition mediated by methylated 642A is not further studied, and there are not comments about this.

Related to the implications of overall viral genome methylation in infected cells, most marks are observed within the coding region of the EV71 RNA (Fig 1B). These viral RNA modifications may alter virus replication directly. This manuscript shows that FBL overexpression promotes methylation of the IRES region (Figs 3 and 4). However, it remains possible that other positions on the viral RNA could be methylated by FBL. I could not find if this possibility was addressed.

Authors focused on viral 3D protein expression as a way to analyze the role of FBL-dependent methylation of host genes. 3D expression in uninfected cells, using VP1 in parallel, revealed transcriptional upregulation of four genes (FOSL1, ITGB3, KCNQ4, and TFPI2). Authors claim that expression of these genes is associated with limited 3D protein synthesis upon FBL-mediated methylation of the IRES element (Fig 5). This conclusion is not directly supported by the results. The limited expression of other non-structural proteins, with potential effect on antiviral response, could also be responsible for this effect.

Reviewer #3: (No Response)

**********

**Part III – Minor Issues: Editorial and Data Presentation Modifications**

Please use this section for editorial suggestions as well as relatively minor modifications of existing data that would enhance clarity.

Reviewer #1: 1. Figs. 3G and 4B, G. Could they quantify GFP expression (preferably by measuring fluorescence), as some effects are very small (e.g., Fig. 4G)?

2. In Fig. 4D (but not in Fig. 3F) VP1 migrates as a double band. Why?

Reviewer #2: -

Reviewer #3: Typos:

Line 84 delete ‘our’

line 431 – should be poly not ploy

line 448 – capitalization

Formatting 117-121

**********

PLOS authors have the option to publish the peer review history of their article (what does this mean?). If published, this will include your full peer review and any attached files.

If you choose “no”, your identity will remain anonymous but your review may still be made public.

**Do you want your identity to be public for this peer review?**  For information about this choice, including consent withdrawal, please see our Privacy Policy.

Reviewer #1: No

Reviewer #2: No

Reviewer #3: No

[NOTE: If reviewer comments were submitted as an attachment file, they will be attached to this email and accessible via the submission site. Please log into your account, locate the manuscript record, and check for the action link "View Attachments". If this link does not appear, there are no attachment files.]

**Figure resubmission:**

-->While revising your submission, we strongly recommend that you use PLOS’s NAAS tool (https://ngplosjournals.pagemajik.ai/artanalysis) to test your figure files. NAAS can convert your figure files to the TIFF file type and meet basic requirements (such as print size, resolution), or provide you with a report on issues that do not meet our requirements and that NAAS cannot fix.

After uploading your figures to PLOS’s NAAS tool - https://ngplosjournals.pagemajik.ai/artanalysis, NAAS will process the files provided and display the results in the "Uploaded Files" section of the page as the processing is complete. If the uploaded figures meet our requirements (or NAAS is able to fix the files to meet our requirements), the figure will be marked as "fixed" above. If NAAS is unable to fix the files, a red "failed" label will appear above. When NAAS has confirmed that the figure files meet our requirements, please download the file via the download option, and include these NAAS processed figure files when submitting your revised manuscript.

**Reproducibility:**

To enhance the reproducibility of your results, we recommend that authors of applicable studies deposit laboratory protocols in protocols.io, where a protocol can be assigned its own identifier (DOI) such that it can be cited independently in the future. Additionally, PLOS ONE offers an option to publish peer-reviewed clinical study protocols. Read more information on sharing protocols at https://plos.org/protocols?utm_medium=editorial-email&utm_source=authorletters&utm_campaign=protocols

# Author comment: Author response to Decision Letter 1

**DOI:** 10.1371/journal.ppat.1014455.r002

17 Jun 2026

Attachment Submitted filename: Response-0612-2.docx

# Peer review: Decision Letter 1

**DOI:** 10.1371/journal.ppat.1014455.r003

**Authors:** Alexander Gorbalenya, Peter Sarnow

3 Jul 2026

PPATHOGENS-D-26-00505R1

Fibrillarin-mediated 2'-O-methylation serves as a translation brake on uncapped enterovirus RNA

PLOS Pathogens

Dear Dr. Liu,

Thank you for submitting your manuscript to PLOS Pathogens. After careful consideration, we feel that it has merit but does not fully meet PLOS Pathogens's publication criteria as it currently stands. Therefore, we invite you to submit a revised version of the manuscript that addresses the points raised during the review process.

In particular, please incorporate the minor issue raised by reviewer #1.

Please submit your revised manuscript by Sep 01 2026 11:59PM. If you will need more time than this to complete your revisions, please reply to this message or contact the journal office at plospathogens@plos.org. When you're ready to submit your revision, log on to https://www.editorialmanager.com/ppathogens/ and select the 'Submissions Needing Revision' folder to locate your manuscript file.

Please include the following items when submitting your revised manuscript:

* A letter that responds to each point raised by the editor and reviewer(s). You should upload this letter as a separate file labeled 'Response to Reviewers'. This file does not need to include responses to any formatting updates and technical items listed in the 'Journal Requirements' section below.

* A marked-up copy of your manuscript that highlights changes made to the original version. You should upload this as a separate file labeled 'Revised Manuscript with Track Changes'.

* An unmarked version of your revised paper without tracked changes. You should upload this as a separate file labeled 'Manuscript'.

If you would like to make changes to your financial disclosure, competing interests statement, or data availability statement, please make these updates within the submission form at the time of resubmission. Guidelines for resubmitting your figure files are available below the reviewer comments at the end of this letter.

As the corresponding author, your ORCID iD is verified in the submission system and will appear in the published article. PLOS supports the use of ORCID, and we encourage all coauthors to register for an ORCID iD and use it as well. Please encourage your coauthors to verify their ORCID iD within the submission system before final acceptance, as unverified ORCID iDs will not appear in the published article. Only the individual author can complete the verification step; PLOS staff cannot verify ORCID iDs on behalf of authors.

We look forward to receiving your revised manuscript.

Kind regards,

Peter Sarnow

Academic Editor

PLOS Pathogens

Alexander Gorbalenya

Section Editor

PLOS Pathogens

Sumita Bhaduri-McIntosh

Editor-in-Chief

PLOS Pathogens

orcid.org/0000-0003-2946-9497

Michael Malim

Editor-in-Chief

PLOS Pathogens

orcid.org/0000-0002-7699-2064

**Additional Editor Comments (if provided):**

**Journal Requirements:**

If the reviewer comments include a recommendation to cite specific previously published works, please review and evaluate these publications to determine whether they are relevant and should be cited. There is no requirement to cite these works unless the editor has indicated otherwise.

1) In the online submission form, you indicated that All sequencing data generated in this study have been deposited in the National Center for Biotechnology Information’s Gene Expression Omnibus (GEO) under the accession number GSE312869. All other relevant data, including primer lists, siRNA sequences, and experimental protocols, are available in the Methods or from the corresponding authors upon reasonable request.

Reviewer access token: qnwhieqczfsxryn

To review GEO accession GSE312869:

Go to https://www.ncbi.nlm.nih.gov/geo/query/acc.cgi?acc=GSE312869

Enter token qnwhieqczfsxryn into the box. All PLOS journals now require all data underlying the findings described in their manuscript to be freely available to other researchers, either

1. In a public repository

2. Within the manuscript itself

3. Uploaded as supplementary information.

This policy applies to all data except where public deposition would breach compliance with the protocol approved by your research ethics board. If your data cannot be made publicly available for ethical or legal reasons (e.g., public availability would compromise patient privacy), please explain your reasons by return email and your exemption request will be escalated to the editor for approval. Your exemption request will be handled independently and will not hold up the peer review process, but will need to be resolved should your manuscript be accepted for publication. One of the Editorial team will then be in touch if there are any issues.

**Reviewers' Comments:**

Reviewer's Responses to Questions

**Part I - Summary**

Please use this section to discuss strengths/weaknesses of study, novelty/significance, general execution and scholarship.

Reviewer #1: The manuscript by Zhang et al. has been substantially improved, and together with the rebuttal letter, fully addresses my concerns. While highlighting the importance of 2’-O-methylation (Nm) at 307C of EV71 RNA by FBL in suppressing viral translation, the authors now acknowledge the potential role of other Nm marks, specifically those within the coding region, in this phenomenon. Evidence is presented that limited production of the viral polymerase 3D prevents upregulation of several pro-viral host genes, thereby impairing viral replication.

Reviewer #2: The authors have satisfactorily addressed most of my concerns

**********

**Part II – Major Issues: Key Experiments Required for Acceptance**

Please use this section to detail the key new experiments or modifications of existing experiments that should be absolutely required to validate study conclusions.

Generally, there should be no more than 3 such required experiments or major modifications for a "Major Revision" recommendation. If more than 3 experiments are necessary to validate the study conclusions, then you are encouraged to recommend "Reject".

Reviewer #1: There are no major issues.

Reviewer #2: (No Response)

**********

**Part III – Minor Issues: Editorial and Data Presentation Modifications**

Please use this section for editorial suggestions as well as relatively minor modifications of existing data that would enhance clarity.

Reviewer #1: The authors suggest that the FBL-mediated attenuation of viral infection is due to the prevention of 3D-dependent upregulation of pro-viral host genes. However, the reduced efficiency of viral infection might also result from the general inhibition of viral protein synthesis. This should be mentioned in the Discussion.

Reviewer #2: (No Response)

**********

PLOS authors have the option to publish the peer review history of their article (what does this mean?). If published, this will include your full peer review and any attached files.

If you choose “no”, your identity will remain anonymous but your review may still be made public.

**Do you want your identity to be public for this peer review?**  For information about this choice, including consent withdrawal, please see our Privacy Policy.

Reviewer #1: No

Reviewer #2: No

[NOTE: If reviewer comments were submitted as an attachment file, they will be attached to this email and accessible via the submission site. Please log into your account, locate the manuscript record, and check for the action link "View Attachments". If this link does not appear, there are no attachment files.]

**Figure resubmission:**

-->While revising your submission, we strongly recommend that you use PLOS’s NAAS tool (https://ngplosjournals.pagemajik.ai/artanalysis) to test your figure files. NAAS can convert your figure files to the TIFF file type and meet basic requirements (such as print size, resolution), or provide you with a report on issues that do not meet our requirements and that NAAS cannot fix.

After uploading your figures to PLOS’s NAAS tool - https://ngplosjournals.pagemajik.ai/artanalysis, NAAS will process the files provided and display the results in the "Uploaded Files" section of the page as the processing is complete. If the uploaded figures meet our requirements (or NAAS is able to fix the files to meet our requirements), the figure will be marked as "fixed" above. If NAAS is unable to fix the files, a red "failed" label will appear above. When NAAS has confirmed that the figure files meet our requirements, please download the file via the download option, and include these NAAS processed figure files when submitting your revised manuscript.

**Reproducibility:**

To enhance the reproducibility of your results, we recommend that authors of applicable studies deposit laboratory protocols in protocols.io, where a protocol can be assigned its own identifier (DOI) such that it can be cited independently in the future. Additionally, PLOS ONE offers an option to publish peer-reviewed clinical study protocols. Read more information on sharing protocols at https://plos.org/protocols?utm_medium=editorial-email&utm_source=authorletters&utm_campaign=protocols

# Author comment: Author response to Decision Letter 2

**DOI:** 10.1371/journal.ppat.1014455.r004

6 Jul 2026

Attachment Submitted filename: Response-0703.docx

# Editor report: Decision Letter 2

**DOI:** 10.1371/journal.ppat.1014455.r005

**Authors:** Alexander Gorbalenya, Peter Sarnow

9 Jul 2026

Dear Dr. Liu,

We are pleased to inform you that your manuscript 'Fibrillarin-mediated 2'-O-methylation serves as a translation brake on uncapped enterovirus RNA' has been provisionally accepted for publication in PLOS Pathogens.

Before your manuscript can be formally accepted you will need to complete some formatting changes, which you will receive in a follow up email. A member of our team will be in touch with a set of requests.

Please note that your manuscript will not be scheduled for publication until you have made the required changes, so a swift response is appreciated.

IMPORTANT: The editorial review process is now complete. PLOS will only permit corrections to spelling, formatting or significant scientific errors from this point onwards. Requests for major changes, or any which affect the scientific understanding of your work, will cause delays to the publication date of your manuscript.

Should you, your institution's press office or the journal office choose to press release your paper, you will automatically be opted out of early publication. We ask that you notify us now if you or your institution is planning to press release the article. All press must be co-ordinated with PLOS.

Thank you again for supporting Open Access publishing; we are looking forward to publishing your work in PLOS Pathogens.

Best regards,

Peter Sarnow

Academic Editor

PLOS Pathogens

Alexander Gorbalenya

Section Editor

PLOS Pathogens

Sumita Bhaduri-McIntosh

Editor-in-Chief

PLOS Pathogens

orcid.org/0000-0003-2946-9497

Michael Malim

Editor-in-Chief

PLOS Pathogens

orcid.org/0000-0002-7699-2064

***********************************************************

Reviewer Comments (if any, and for reference):

# Editor report: Acceptance letter

**DOI:** 10.1371/journal.ppat.1014455.r006

**Authors:** Alexander Gorbalenya, Peter Sarnow

Dear Dr. Liu,

We are delighted to inform you that your manuscript, "Fibrillarin-mediated 2'-O-methylation serves as a translation brake on uncapped enterovirus RNA," has been formally accepted for publication in PLOS Pathogens.

We have now passed your article onto the PLOS Production Department who will complete the rest of the pre-publication process. All authors will receive a confirmation email upon publication.

The corresponding author will soon be receiving a typeset proof for review, to ensure errors have not been introduced during production. Please review the PDF proof of your manuscript carefully, as this is the last chance to correct any scientific or type-setting errors. Please note that major changes, or those which affect the scientific understanding of the work, will likely cause delays to the publication date of your manuscript. Note: Proofs for Front Matter articles (Pearls, Reviews, Opinions, etc...) are generated on a different schedule and may not be made available as quickly.

Soon after your final files are uploaded, the early version of your manuscript, if you opted to have an early version of your article, will be published online. The date of the early version will be your article's publication date. The final article will be published to the same URL, and all versions of the paper will be accessible to readers.

For Research Articles, you will receive an invoice from PLOS for your publication fee after your manuscript has reached the completed accept phase. If you receive an email requesting payment before acceptance or for any other service, this may be a phishing scheme. Learn how to identify phishing emails and protect your accounts at https://explore.plos.org/phishing.

Thank you again for supporting open-access publishing; we are looking forward to publishing your work in PLOS Pathogens.

Best regards,

Sumita Bhaduri-McIntosh

Editor-in-Chief

PLOS Pathogens

orcid.org/0000-0003-2946-9497

Michael Malim

Editor-in-Chief

PLOS Pathogens

orcid.org/0000-0002-7699-2064
